# Epigenetic traits inscribed in chromatin accessibility in aged hematopoietic stem cells

**DOI:** 10.1038/s41467-022-30440-2

**Published:** 2022-05-16

**Authors:** Naoki Itokawa, Motohiko Oshima, Shuhei Koide, Naoya Takayama, Wakako Kuribayashi, Yaeko Nakajima-Takagi, Kazumasa Aoyama, Satoshi Yamazaki, Kiyoshi Yamaguchi, Yoichi Furukawa, Koji Eto, Atsushi Iwama

**Affiliations:** 1 Department of Cellular and Molecular Medicine, Graduate School of Medicine, Chiba University, Chiba, Japan; 2 Division of Stem Cell and Molecular Medicine, Center for Stem Cell Biology and Regenerative Medicine, The Institute of Medical Science, The University of Tokyo, Tokyo, Japan; 3 Department of Regenerative Medicine, Chiba University Graduate School of Medicine, Chiba, Japan; 4 Division of Stem Cell Biology, Center for Stem Cell Biology and Regenerative Medicine, The Institute of Medical Science, The University of Tokyo, Tokyo, Japan; 5 Laboratory of Stem Cell Therapy, Faculty of Medicine, University of Tsukuba, Ibaraki, Japan; 6 Division of Clinical Genome Research, Advanced Clinical Research Center, Institute of Medical Science, The University of Tokyo, Tokyo, Japan; 7 Department of Clinical Application, Center for iPS Cell Research and Application, Kyoto University, Kyoto, Japan; 8 Laboratoty of Cellular and Molecular Chemistry, Graduate School of Pharmaceutical Sciences, The University of Tokyo, Tokyo, Japan

**Keywords:** Stem-cell research, Myelopoiesis, Haematopoietic stem cells, Ageing

## Abstract

Hematopoietic stem cells (HSCs) exhibit considerable cell-intrinsic changes with age. Here, we present an integrated analysis of transcriptome and chromatin accessibility of aged HSCs and downstream progenitors. Alterations in chromatin accessibility preferentially take place in HSCs with aging, which gradually resolve with differentiation. Differentially open accessible regions (open DARs) in aged HSCs are enriched for enhancers and show enrichment of binding motifs of the STAT, ATF, and CNC family transcription factors that are activated in response to external stresses. Genes linked to open DARs show significantly higher levels of basal expression and their expression reaches significantly higher peaks after cytokine stimulation in aged HSCs than in young HSCs, suggesting that open DARs contribute to augmented transcriptional responses under stress conditions. However, a short-term stress challenge that mimics infection is not sufficient to induce persistent chromatin accessibility changes in young HSCs. These results indicate that the ongoing and/or history of exposure to external stresses may be epigenetically inscribed in HSCs to augment their responses to external stimuli.

## Abstract (web summary)

Haematopoietic stem cells (HSCs) exhibit considerable cell-intrinsic changes with age. Here the authors demonstrate that differentially accessible regions in aged HSC chromatin are enriched for stress-responsive enhancers and act as an epigenetic hub to augment transcriptional responses of aged HSCs to external stimuli.

## Introduction

Hematopoietic stem cells (HSCs) give rise to all types of blood cells throughout life and show considerable changes with age, such as impaired regenerative potential, myeloid/platelet-biased differentiation, and low production of lymphocytes^1–4^. The HSC niche, which is essential for the maintenance of HSCs, also undergoes considerable changes with age^5–7^. Aged HSCs exhibit cell-intrinsic molecular changes that cannot be restored by a young niche, as suggested by transplantation of aged HSCs into lethally irradiated young recipient mice^8^. We showed that aged HSCs engrafted in the intact young bone marrow (BM) niche in non-conditioned recipient mice had sustained functional defects. The young intact niche largely restored the transcriptional profile of aged HSCs, but not their DNA methylation profiles, highlighting a key role for altered epigenome in HSC aging^9^.

HSC aging is also associated with an increased risk of hematological malignancies^2,4^. The onset of representative age-associated myeloid malignancies, such as myelodysplastic syndromes (MDS) and myeloproliferative neoplasms (MPN), is highly age-dependent. Epigenetic modifier genes, such as *DNMT3A*, *TET2*, *ASXL1*, and *EZH2*, are frequently mutated in MDS and MPN^10–12^. A dysregulated function of these epigenetic regulators induces the reprogramming of DNA and/or histone modifications, leading to the induction of MDS or MPN-like diseases in mice^13–15^. Of note, recurrent somatic mutations with clonal hematopoiesis have been identified in healthy elderly individuals using comprehensive genome sequencing^16,17^. It is called age-related clonal hematopoiesis (ARCH) or clonal hematopoiesis of indeterminate potential (CHIP)^18,19^. *DNMT3A*, *TET2*, and *ASXL1* mutations are also frequent in CHIP^16–19^. Individuals with CHIP mutations have a higher risk of hematological malignancies. These findings strongly suggest that epigenetic factors also mediate HSC aging and age-associated hematological malignancies.

Various epigenetic alterations have been recognized as a hallmark of aging^20^. Epigenetic markers can readily change over time, and this “epigenetic drift”, which depends on both intrinsic and extrinsic factors, may play an important role in HSC aging^21^. Proliferation-dependent alterations in the DNA methylation are observed during HSC aging, occurring in regions associated with lineage potential and selectively target genes exclusively expressed downstream of HSCs or expressed at higher levels in progenitor and/or effector cells relative to HSCs^22^. Genome-wide comparisons of histone modifications between young and aged mouse HSCs have provided potential mechanisms that contribute to HSC aging^23^. Profiling of histone modifications also deciphered significant alterations in the regulation of enhancers and transcription factors during human HSC aging^24^. In contrast with epigenomic analyses, chromatin accessibility reflects a comprehensive network of enhancers, promoters, and transcription factors, which have critical roles in normal hematopoiesis and cancer development^25^. However, changes in chromatin accessibility in HSCs have not yet been clarified during aging.

In this work, we present an integrated analysis of transcriptome and chromatin accessibility of aged HSCs and progenitors. Transposase-accessible chromatin sequencing (ATAC-seq) assays reveal that alterations in chromatin accessibility preferentially take place in HSCs with aging, which gradually resolve with differentiation. Differentially open accessible regions in aged HSCs are enriched for enhancers with binding motifs of stress-responsive transcription factors and are associated with augmented gene expression upon stresses in aged HSCs, suggesting that the ongoing and/or history of exposure to external stresses may be epigenetically inscribed in HSCs to augment their responses to external stimuli. However, a short-term stress challenge that mimics infection is not sufficient to induce persistent chromatin accessibility changes in young HSCs.

## Results

### Altered transcriptome of HSPCs during aging

**Fig. 1:**
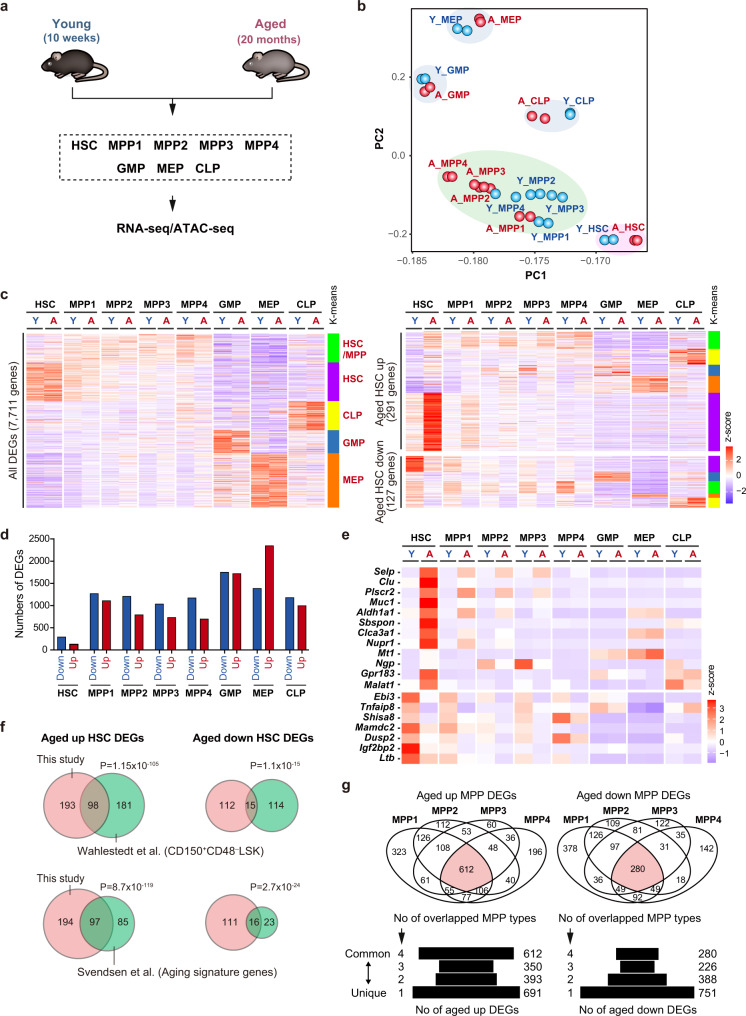
Altered transcriptome of hematopoietic stem and progenitor cells during aging. **a** Experimental strategy. Bone marrow cells from 10-week-old mice (Young) and 20-month-old mice (Aged) were pooled and sorted in duplicate. **b** PCA plot based on the z-scores of expression values (DESeq2 normalized counts) in each fraction. Blue and red circles indicate young and aged cells (*n* = 2). **c** Heatmaps showing the z-scores of expression values (DESeq2 normalized counts) in all DEGs (7711 genes, left panel) and DEGs in HSCs (291 Aged HSC up DEGs and 127 Aged HSC down DEGs, right panel). Genes are divided into five clusters [HSC (purple), HSC/MPP (green), GMP (blue), MEP (orange), and CLP (yellow)-type] based on K-means clustering. **d** The numbers of up and down DEGs in aged cells in each fraction. **e** Heatmap showing the z-scores of expression values (DESeq2 normalized counts) of representative DEGs in aged HSCs. **f** Venn diagram displaying the numbers of common DEGs in aged HSCs between this study and reported studies^28,29^. *P* values by Fisher’s exact test are indicated. **g** The numbers of DEGs common among MPP fractions. Venn diagrams (upper panels). Pyramid plots (lower panels), in which genes at the top and bottom were shared by 4 MPP types and unique to one of the 4 MPP types, respectively. Source data are provided as a Source Data file.

To investigate the intrinsic changes in hematopoietic stem and progenitor cells (HSPCs) during aging, we purified HSCs, multipotent progenitor (MPP) 1, megakaryocyte/erythroid-biased MPP2, myeloid-biased MPP3, lymphoid-primed MPP4, granulocyte-macrophage progenitors (GMPs), megakaryocyte-erythrocyte progenitors (MEPs), and common lymphoid progenitors (CLPs) from 10-week-old (Young) and 20-month-old aged (Aged) mice and performed RNA-seq and ATAC-seq analyses^26,27^ (Fig. 1a). Principal component analysis (PCA) of RNA-seq data revealed that aged HSPCs show different transcriptomic profiles from young HSPCs, but the difference was mild (Fig. 1b). Comparison of the transcriptomes of young and aged counterparts revealed various differentially expressed genes (DEGs) in each fraction (FDR *q* < 0.01) (Fig. 1c, Supplementary Fig. 1, and Supplementary Data 1), among which HSCs had a much lower number of DEGs than the progenitor fractions (Fig. 1d). The majority of DEGs in HSCs were also differentially expressed in the MPP fractions, albeit to a lesser extent, while oligopotent progenitors showed entirely unique changes in the transcriptome with aging. (Fig. 1c, d, and Supplementary Fig. 1). The hallmark genes of aged HSCs, such as *P-selectin* (*Selp*) and *Clusterin* (*Clu*)^23,28–30^ were markedly upregulated in aged HSCs (Fig. 1e, Supplementary Fig. 1, and Supplementary Data 1). A large portion of aged HSC DEGs were shared with those in aged CD150^+^CD48^–^LSK HSCs^23,29^ and Aging signature genes which were consistently detected among 16 published and unpublished datasets^28^ (Fig. 1f, Supplementary Data 1), indicating that the transcriptome changes during HSC aging have certain trends and reproducibility among different experiments. The transcriptome of downstream progenitors also showed a unique pattern of changes during aging (Fig. 1b, c). Notably, the transcriptome changes during aging were more drastic in MPPs and oligopotent progenitors than in HSCs (Fig. 1d and Supplementary Fig. 1), among which a large portion of DEGs were common among MPP1-4 (Fig. 1g, Supplementary Data 1).

Gene ontology (GO) analysis revealed that upregulated genes in aged MPPs were associated with the metabolic process, cell cycle, protein folding, and RNA splicing, including *Idh2, Aurka, Aurkb, Calr*, and *Srsf2*, while downregulated genes in aged MPPs included key hematopoietic transcription factor genes, such as *Lmo2, Runx2, Irf5*, and *Notch1* (Supplementary Fig. 2a and Supplementary Data 2). Genes associated with cell cycle and cell division were significantly upregulated in aged MPPs and GMP, while they were downregulated in aged CLPs (Supplementary Fig. 2a). Homer motif analysis, which yields the binding motifs of transcription factors at the promoters of DEGs, revealed that IRF target genes were significantly enriched in upregulated genes in aged oligopotent progenitors, which may reflect age-associated inflammation in the BM (Supplementary Fig. 2b, Supplementary Data 3). E2F target genes were significantly enriched in downregulated genes in aged CLPs, which corresponded well with the negative association of cell cycle and cell division GO terms in aged CLPs (Supplementary Fig. 2b). These results suggested de-regulated cell cycle in aged HSPCs. To confirm these transcriptomic results, we directly investigated the cell-cycle state of young and aged HSPCs (Supplementary Fig. 3a). Short-term in vivo 5-ethynyl-2′-deoxyuridine (EdU) incorporation assays revealed that the cell cycle was significantly promoted in aged MPPs including MPP4, which is consistent with our present GO data as well as previous reports^31^. In contrast, the cell cycle of CLPs was significantly perturbed with aging as expected from the negative enrichment of E2F at the promoters of DEGs. A decreased output of lymphoid cells is one of the major aging phenotypes in hematopoiesis. Our data suggest that an impaired proliferation of aged CLPs may partially contribute to the reduction in their number (Supplementary Fig. 3b) and a decreased output of lymphoid cells.

### Characteristics of chromatin accessibility in aged HSPCs

**Fig. 2:**
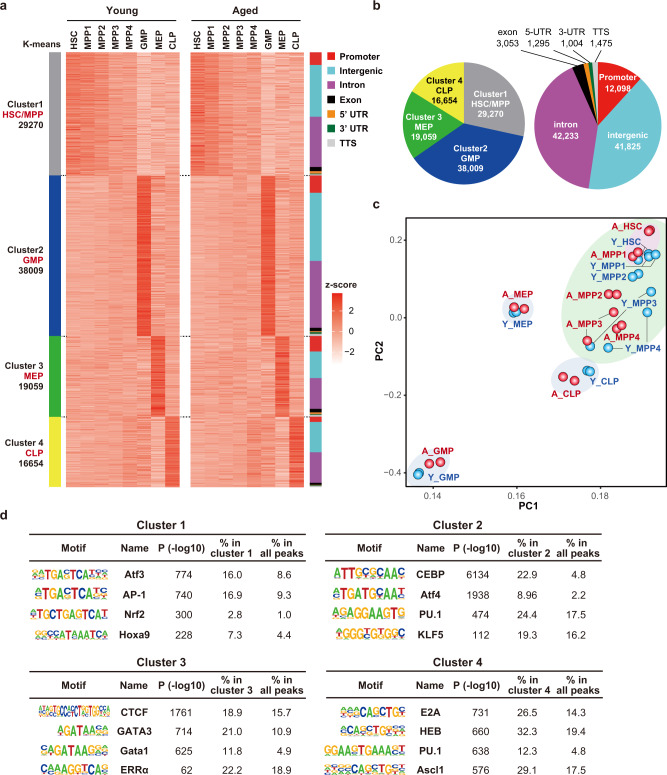
Characteristics of chromatin accessibility in aged HSPCs. **a** Heatmap showing the z-scores of ATAC count values (DESeq2 normalized counts) in all 102,992 peaks in young and aged HSPCs. Peaks were divided into 4 clusters using K-means clustering. Cluster 1 showed higher accessibility in HSCs and MPPs than the downstream progenitors. Clusters 2, 3, and 4 showed the highest accessibility in GMPs, MEPs, and CLPs, respectively. Right bar indicates the annotations of distribution on the genome. **b** Pie charts showing the percentage of each cluster subtype (left) and annotation (right) of all accessible peaks. **c** PCA plot based on the ATAC count values in log2 (DESeq2 normalized counts) in each fraction. Blue and red circles indicate young and aged cells (*n* = 2). **d** Homer motif analysis of accessible peaks in each cluster. Top 4 motifs and −log10 *p* values are depicted. The enrichment of each motif in the peaks in each cluster and the background peaks are indicated. All peaks, which includes 102,992 peaks from all fractions, were used as background. Hypergeometric tests were performed to calculate *p* values. No adjustments were made for multiple comparisons.

We next investigated chromatin accessibility in young and aged HSCs. Accumulating evidence has emerged regarding the role of aberrant chromatin accessibility in aged hematopoietic cells and hematological malignancies^25,32,33^, while chromatin accessibility in aged HSPCs has not yet been investigated. We profiled the accessibility landscapes of HSCs, MPP1, MPP2, MPP3, MPP4, GMPs, MEPs, and CLPs purified from young and aged mice using ATAC-seq. A total of 102,992 accessible peaks were detected in young and aged HSPCs, respectively (Fig. 2a). A total of 12,098 peaks were localized at the promoter regions. Peaks in the non-promoter region were mainly located in intergenic or intron regions, that generally represent active or poised enhancers [41,825 at intergenic, 42,233 at intron, 3053 at exon, 1295 at 5′-untranslated regions (UTR), 1004 at 3′-UTR, and 1475 at the transcription termination site (TTS)] (Fig. 2b). PCA of ATAC-seq data revealed that aged HSPCs show different profiles of chromatin accessibility from those of young HSPCs, but the difference was mild as the transcriptomic differences (Fig. 2c). K-means clustering clearly classified these peaks into four distinct clusters based on the accessibility in each fraction. Cluster 1 (29,270 peaks) showed a higher accessibility in HSCs and MPPs than in the downstream progenitors. Clusters 2, 3, and 4 showed the highest accessibility in GMPs (380,09 peaks), MEPs (19,059 peaks), and CLPs (16,654 peaks), in this order (Fig. 2a). DNA motif analysis of ATAC peaks identified a significant enrichment of the binding motif of Hoxa9, an important transcription factor for HSPC maintenance, in Cluster 1. Clusters 2, 3, and 4 showed an enrichment of motifs for CEBP, GATA, and E2A, key transcriptional regulators in GMPs, MEPs, and CLPs, respectively (Fig. 2d, Supplementary Data 4). These results were consistent with previous observations that each individual cell type utilizes different accessible regions to control differentiation^25,32^.

### Differentially accessible regions in aged HSPCs

**Fig. 3:**
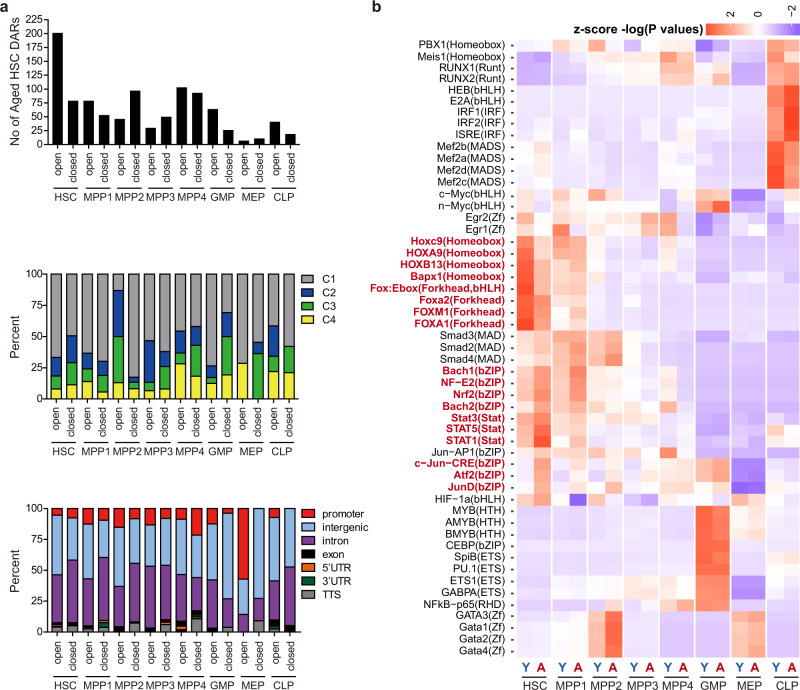
Differentially accessible regions in aged HSPCs. **a** The number of DARs in aged HSPCs compared with their counterparts (upper panel). Percentages of each cluster subtype based on clustering in Fig. 2a (middle panel) and annotation (lower panel) of all DARs. DARs were calculated using DESeq2 using generalized linear model and a cutoff *p* < 0.001 was used to define the DARs. No adjustments were made for multiple comparisons. **b** Heatmap showing the enrichment score [z-scores of −log (*P* values)] of the transcription factor binding motifs at accessible peaks detected in ATAC-seq in each young and aged HSPC fraction. *P* values were derived from Hypergeometric tests of motif analysis. No adjustments were made for multiple comparisons. Source data are provided as a Source Data file.

Next, we compared chromatin accessibility between young and aged counterparts in each fraction. A total of 281 differentially accessible regions (DARs) were identified between young and aged HSCs using DEseq2 and biological duplicates (*n* = 2). The 201 and 80 DARs were open and closed in aged HSCs (*p* < 0.001), respectively (Supplementary Data 5a). Among the HSPC fractions, HSCs had the largest number of DARs, and the differences in chromatin accessibility between young and aged cells tended to decrease during differentiation (Fig. 3a, Supplementary Data 5a–h). This suggested that the alterations in chromatin accessibility preferentially occurred in HSCs, the cells that are located at the top of the hematopoietic hierarchy. The DAR profile is quite different from that of DEGs, which is much more frequent in progenitors than in HSCs. Most DARs were concentrated in Cluster 1, which had high accessibility in HSCs and MPPs, and were mostly located in intergenic and intronic regions (Fig. 3a). We then compared the frequencies of the transcription factor binding motifs at accessible peaks detected in each fraction with those in the background peaks using hypergeometric distribution of Homer software and calculated the *p* values of enrichment. The combined peaks detected in all fractions were used for the background to remove imbalance in the sequence content. Assuming that −log (*P* value) reflects the abundance of motif sequences in each fraction, we created a heatmap based on this value. Z-scoring was performed in the final step to clarify the contrast between each fraction. The motifs used in the figure are arbitrarily selected as they are known to be important for hematopoietic function and/or differentiation. This motif analysis revealed that transcriptional regulatory regions with ATF family (c-Jun-CRE, Atf) motifs, STAT family (Stat1, Stat3, Stat5) motifs, and CNC family (Nrf2, NF-E2, Bach1, Bach2) motifs tended to become open in aged HSCs, while those with Hox and FOX family (Hoxa9, Hoxc9, Foxa1, Foxa2) motifs were closed in aged HSCs (Fig. 3b). Transcriptional regulatory regions with GATA family (Gata1, Gata2, Gata3) motifs became open in aged MPP2, compared to those in young MPP2 (Fig. 3b).

### Altered chromatin accessibility in aged HSCs

**Fig. 4:**
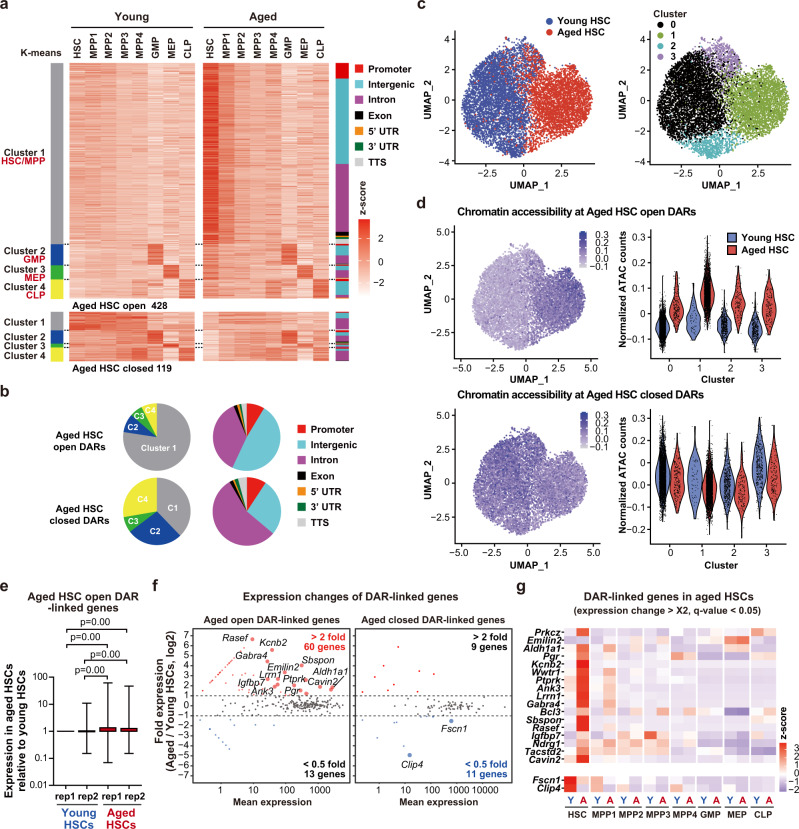
Altered accessibility of transcription factors in aged HSCs. **a** Heatmap showing the z-scores of ATAC count values (DESeq2 normalized counts) in open (upper) and closed (lower) DARs in aged HSCs (4 biological replicates). Peaks were divided into 4 clusters using K-means clustering. Clusters and annotations are shown as in Fig. 2a. **b** Pie charts showing the percentage of each cluster subtype and annotation of open and closed DARs in aged HSCs. **c** UMAP plots illustrating the identification of cell clusters based on single cell ATAC peak profiling of young and aged HSCs. **d** Feature plots (left panels) and violin (right panels) plots showing the average chromatin accessibilities (normalized ATAC peak counts per million) at Aged HSC open (upper panels) and closed (lower panels) DARs. Average accessibilities were calculated using AddModuleScore function of Seurat. **e** Box plots showing fold expression of Aged HSC open DAR-linked genes in young and aged HSCs. Expression of DAR-linked genes is indicated as a fold expression relative to that in young HSCs (replication #1) using normalized read counts (DESeq2 normalized counts + 1). In total 381 Aged HSC open DAR-linked genes were examined in two biologically independent samples. Data derived from a single experiment are depicted. Boxes represent the 25–75 percentile ranges with the median of horizontal line. The ends of vertical lines represent minimum or maximum values. Paired Student’s one-tailed *t* tests were performed to calculate *p* values. **f** MA plots showing expression of DAR-linked genes in aged HSCs relative to that in young HSCs. The pink and blue dots represent up- and downregulated DAR-linked genes greater than twofold during aging, respectively. **g** Heatmap showing the z-scores of expression values (DESeq2 normalized counts) of representative DAR-linked genes that showed expression changes greater than twofold with *q* values <0.05 in aged HSCs. The distance between DARs and the nearest genes is listed in Supplementary Data 5i. Source data are provided as a Source Data file.

Because HSCs are the major cells that show an altered chromatin accessibility, aging of the hematopoietic system could be largely attributed to the alterations in HSCs with aging. We therefore investigated the differences in chromatin accessibility between young and aged HSCs in more detail using four biological replicates. With improved sensitivity and statistical significance, we identified 428 and 119 open and closed DARs in aged HSCs, respectively (FDR *q* < 0.01) (Fig. 4a, Supplementary Data 5i). Most DARs were in non-promoter regions and assigned to Cluster 1, which showed a higher accessibility in HSCs and MPPs than oligopotent progenitors (Fig. 4a, b). The aged HSCs had four times more open DARs than closed DARs. Differential accessibilities in aged HSCs were gradually resolved during differentiation, particularly in MPP2, leaving significant, albeit moderate, differences in the MPP fraction and oligopotent progenitors (Supplementary Fig. 4).

To further elucidate the chromatin accessibility changes at single cell levels, we performed single cell ATAC sequencing (scATAC-seq) analysis using young and aged HSCs. After quality control, we used the data from 5,002 young and 3,520 aged single HSCs and identified 4 major clusters based on dimension reduction by UMAP (Fig. 4c). Young and aged HSCs were mostly assigned to Clusters 0/2 and Cluster 1, respectively, while a part of young and aged HSCs were assigned to the same cluster, Cluster 3 (Fig. 4c). Of note, chromatin accessibilities at Aged HSC open and closed DARs were significantly increased and decreased, respectively, in all clusters, indicating that altered chromatin accessibility identified in ATAC-seq of bulk HSCs are conserved among most HSCs at single cell levels (Fig. 4d).

To assess the correlation between changes in chromatin accessibility and transcription, DARs were connected to the nearest genes based on their distance to TSSs. The genes linked to Aged HSC open DARs showed significantly higher levels of expression in aged HSCs than young HSCs (Fig. 4e). Among 381 and 101 genes linked to Aged HSC open and closed DARs, respectively, 60 Aged HSC open genes and 11 Aged HSC closed genes showed up- and downregulation, respectively, greater than twofold in aged HSCs relative to young HSCs (Fig. 4f). Assuming that the genes nearest to DARs are not always the real target of transcriptional regulatory regions at DARs, these results indicate that a significant portion of DARs accounts for differential expression between young and aged HSCs. Of interest, the differential expression of representative DAR-linked genes, which showed expression changes greater than twofold with *q* values < 0.05 in aged HSCs, were sharply resolved upon differentiation (Fig. 4g).

### Altered accessibility of transcription factors in aged HSCs

**Fig. 5:**
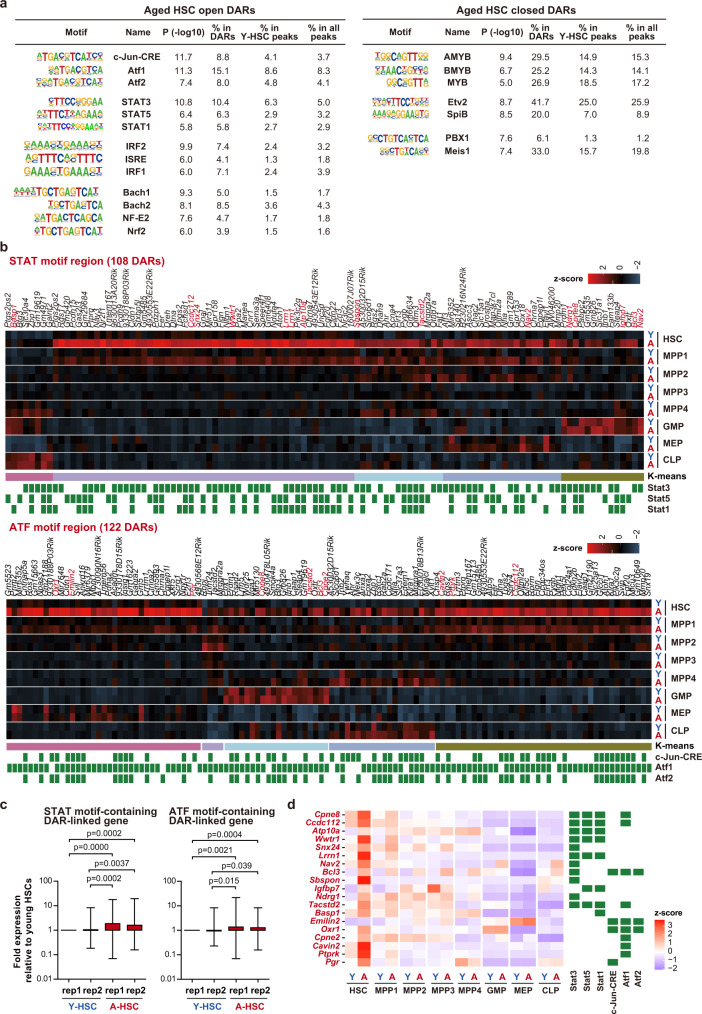
Enrichment of transcription factor binding motifs at DARs in aged HSCs. **a** Motif analysis of open and closed DARs in aged HSCs. Motifs and −log10 *p* values are depicted. The enrichment of each motif in DARs in aged HSCs, the peaks in young HSCs, and the background peaks are indicated. All peaks, which includes 102,992 peaks from all fractions, were used as background. *P* values were derived from Hypergeometric tests of motif analysis. No adjustments were made for multiple comparisons. **b** Heatmap showing the z-scores of ATAC counts (DESeq2 normalized counts) in STAT (upper panel) and ATF motif (lower panel) regions of open DARs in aged HSCs. Regions were divided into 5 clusters using K-means clustering. The distance between DARs and the nearest genes is listed in Supplementary Data 5i. The genes that showed a significant upregulation with *q* values <0.05 in aged HSCs in expression are indicated in red. **c** Box plots showing expression of the genes linked to Aged open DARs containing STAT or ATF motifs. Expression of DAR-linked genes in aged HSCs is indicated as a fold expression relative to that in young HSCs (replication #1) using normalized read counts (DESeq2 normalized counts + 1). STAT-motif and ATF-motif-containing DAR-linked genes (103 and 116 genes, respectively) were examined two biologically independent samples. Data derived from a single experiment are depicted. Boxes represent the 25–75 percentile ranges with the median of horizontal line. The ends of vertical lines represent minimum or maximum values. ns not significant by the Paired Student’s one-tailed *t* tests were performed to calculate *p* values. **d** Heatmap showing the expression values (normalized DESeq2 counts) of genes that showed a significant upregulation in expression in (**b**). The distance between DARs and the nearest genes is listed in Supplementary Data 5i. Source data are provided as a Source Data file.

To investigate the transcription factors that are recruited to DARs, we analyzed the DNA-binding motifs of transcription factors at DARs. The motifs of the ATF family (c-Jun-CRE, Atf1, and Atf2), STAT family (Stat1, Stat3, Stat5), and CNC family (Nrf2, NF-E2, Bach1, Bach2) were enriched in aged HSC open DARs, while the motifs of the MYB family (Myb, AMyb, BMyb), SpiB, Pbx1, and Meis1 were enriched in aged HSC closed DARs (Fig. 5a, Supplementary Data 5j, k). Because of the smaller numbers of DARs in progenitor fractions, not many motifs were enriched in progenitor DARs compared with HSC DARs (Supplementary Fig. 5, Supplementary Table from 5l to 5y). However, among the motifs enriched in Aged HSC open DARs, motifs of IRF family (IRF1, IRF2, ISRE) were significantly enriched in aged MPP1-4 open DARs, while motifs of ATF family were only moderately enriched in aged MPP1 open DARs. Among the motifs enriched in Aged HSC closed DARs, motifs of MYB family (Myb, AMyb, BMyb) were enriched in aged MPP closed DARs. PBX1 and/or RFX motifs were also enriched in MPP and oligopotent progenitor closed DARs. Although a part of DARs were associated with differential expression of DAR-linked genes between young and aged MPPs, the changes in the expression levels of DAR-linked genes in MPPs tended to be low compared with those in HSCs, particularly with genes linked with open DARs in aged MPP cells (Fig. 4f, Supplementary Fig. 6).

To further characterize the association between transcription factors and DARs, we focused on ATF and STAT binding motifs that showed a markedly high enrichment in aged HSC open DARs. ATF family transcription factors bind to cAMP response element (CRE). ATF2/c-Jun heterodimer also binds to CRE. Among the transcription factors targeting these motifs, Stat3 and c-Jun exhibited transcriptional upregulation in aged HSCs (Supplementary Fig. 7). DARs with STAT or ATF motifs showed an increased accessibility in aged HSCs, which was moderately retained in MPP1, but considerably resolved in downstream progenitors, except for some DARs (Fig. 5b). Among Aged HSC open DARs, the genes linked to DARs containing STAT or ATF motifs showed significantly higher levels of expression in aged HSCs than young HSCs (Fig. 5c). Differential expression was most evident in HSCs but was attenuated in downstream progenitor cells (Fig. 5d).

Several previous reports showed reduced TGF-β signaling in aged HSCs^23,34,35^. However, the TGF-β signaling gene set did not show enrichment in the transcriptome of aged HSCs in this study (Supplementary Fig. 8a). We next compared Aged HSC open and closed DAR-linked genes with KEGG TGF-β signaling gene set, but they barely overlapped to each other (Supplementary Fig. 8b). Smad motifs did not show significant enrichment in Aged HSC DARs, either (Supplementary Data 5j, k). These results correspond well to the recent report by Flohr Svendsen et al.^30^ and indicate that TGF-β signaling is not profoundly involved in HSC aging.

### Augmented response of DAR-linked genes to cytokines

**Fig. 6:**
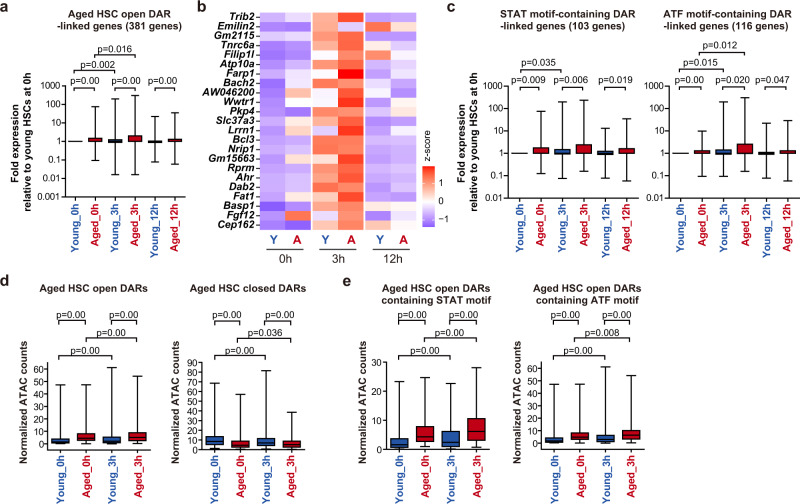
Transcriptional response of DAR-linked genes to cytokine stimulation. **a** Box plots showing fold expression of DAR-linked genes in young and aged HSCs after cytokine stimulation. Young and aged HSCs were stimulated with a mixture of cytokines (SCF, TPO, GM-CSF, IL-1, IL-6, and IL-11) in vitro and subjected to RNA-seq analysis at the indicated time points. Expression of Aged HSC open DAR-linked genes is indicated as a fold expression relative to that in young HSCs (0 h) using normalized read counts (DESeq2 normalized counts + 1). In total 381 Aged HSC open DAR-linked genes were examined. Data derived from a single experiment are depicted. Boxes represent the 25–75 percentile ranges with the median of horizontal line. The ends of vertical lines represent minimum or maximum values. Paired Student’s one-tailed *t* tests were performed to calculate *p* values. **b** Heatmap showing the expression values (DEseq2 normalized counts) of representative genes that showed a significant upregulation greater than threefold (Young 3 h vs. Young 0 h) in (**a**). **c** Box plots showing fold expression of the genes linked to STAT or ATF motif-containing DARs in (**a**). STAT-motif and ATF-motif-containing DAR-linked genes (103 and 116 genes, respectively) were examined. Data derived from a single experiment are depicted. Boxes represent the 25–75 percentile ranges with the median of horizontal line. The ends of vertical lines represent minimum or maximum values. Paired Student’s one-tailed *t* tests were performed to calculate *p* values. **d** Box plots showing normalized ATAC counts (counts per million, CPM) before and after cytokine stimulation of young and aged HSCs at Aged HSC open and closed DARs. Aged open DARs (428) and aged close DARs (119) were examined. Data derived from a single experiment are depicted. Boxes represent the 25–75 percentile ranges with the median of horizontal line. The ends of vertical lines represent minimum or maximum values. Paired Student’s one-tailed *t* tests were performed to calculate *p* values. **e** Box plots showing normalized ATAC counts (CPM) before and after cytokine stimulation of young and aged HSCs at Aged HSC open DARs containing STAT or ATF binding motif. STAT-motif and ATF-motif-containing Aged HSC open DARs (108 and 117 DARs, respectively) were examined. Data derived from a single experiment are depicted. Boxes represent the 25–75 percentile ranges with the median of horizontal line. The ends of vertical lines represent minimum or maximum values. Paired Student’s one-tailed *t* tests were performed to calculate *p* values. Source data are provided as a Source Data file.

Since DARs relaxed in aged HSCs showed enrichment of motifs of transcription factors associated with various stresses including cytokine signaling, we next stimulated young and aged HSCs with a mixture of cytokines (SCF, TPO, GM-CSF, IL-1, IL-6, and IL-11) in vitro to investigate the transcriptional response of Aged HSC open DAR-linked genes by RNA-seq analyses. DAR-linked genes showed significantly higher levels of basal expression in aged HSCs than young HSCs. Cytokine stimulation significantly enhanced the expression of DAR-linked genes in both young and aged HSCs at 3 h post-stimulation, which recovered to basal levels by 12 h, indicating that DAR-linked genes include many early-response genes to cytokine stimulation (Fig. 6a, b, Supplementary Data 6). Notably, DAR-linked genes in aged HSCs reached significantly higher levels of expression than in young HSCs (Fig. 6a, b). DARs containing STAT or ATF motifs showed similar behaviors in expression (Fig. 6c), suggesting that aged HSC open DARs contribute to augmented transcriptional responses under stress conditions.

Differential chromatin accessibilities in aged HSCs were gradually resolved during differentiation, although they were retained in many progenitor fractions at lower levels than HSCs (Supplementary Fig. 4). We then evaluated the expression changes in MPP fractions after cytokine stimulation in genes linked to Aged HSC open DARs (Supplementary Fig. 9). As observed in aged HSCs, the steady-state expression of these genes was slightly elevated in aged MPP1, MPP2, and MPP3, but not in MPP4 compared with young counterparts. After cytokine stimulation, only aged MPP1 showed significantly enhanced upregulation than young MPP1, while aged MPP2, MPP3, and MPP4 showed expression changes comparable to young counterparts. These results clearly indicate that the enhanced cytokine responses of the genes linked to Aged HSC open DARs are lost during differentiation.

To understand the chromatin accessibility changes after cytokine stimulation at DARs in HSCs, we performed ATAC-seq in young and aged HSCs before and after cytokine stimulation. As expected, we found that chromatin accessibilities at Aged HSC open DARs significantly increased after cytokine stimulation in both young and aged HSCs (Fig. 6d). These trends were similarly observed at DARs containing STAT or ATF motifs (Fig. 6e). These results support the notion that DARs in aged HSCs contribute to augmented transcriptional responses under stress conditions. However, the fold increases in ATAC counts at Aged HSC open DARs were moderate compared with fold changes in transcriptome, suggesting that the Aged HSC open DARs represent active or primed enhancers/promoters. Unexpectedly, chromatin accessibilities at Aged closed DARs significantly decreased after cytokine stimulation (Fig. 6d), suggesting that cytokine stimuli are also associated with the formation of a part of closed DARs.

### Altered histone modifications at DARs in aged HSCs

To understand the epigenomic status of DARs in aged HSCs, we first analyzed our previous whole-genome bisulfite sequencing (WGBS) datasets of HSCs from 10-week-old and 20-month-old mice^9^. DNA methylation levels were significantly decreased at Aged HSC open DARs. In contrast, DNA methylation levels showed no significant change at closed DARs (Supplementary Fig. 10).

**Fig. 7:**
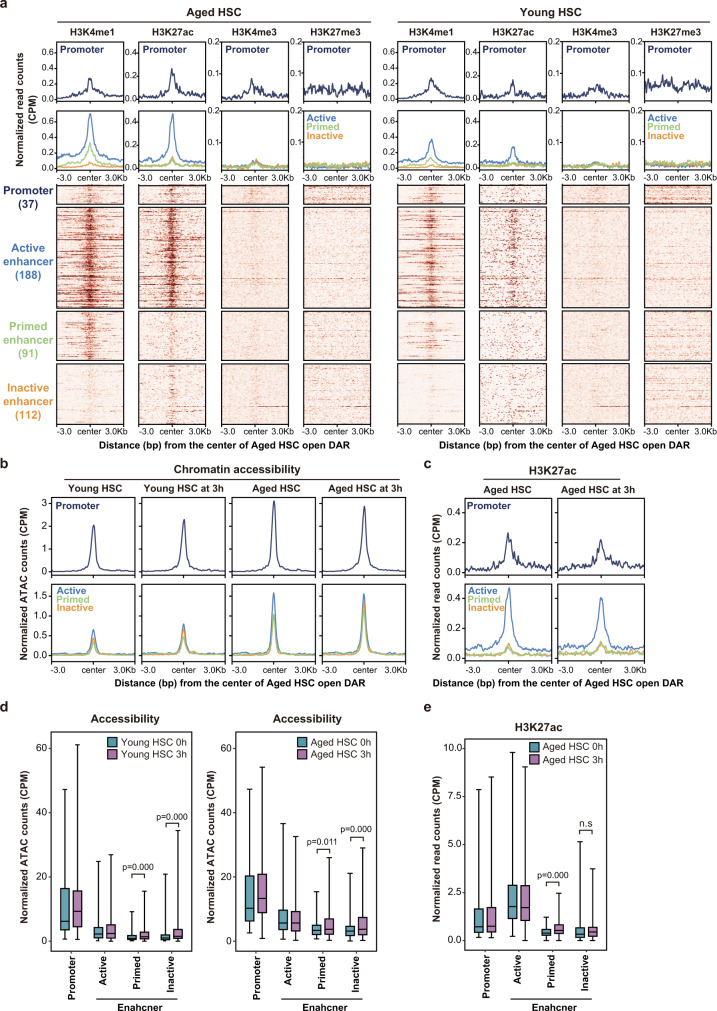
Altered histone modifications at DARs in aged HSCs. **a** Heatmap showing the levels of H3K4me1, H3K27ac, H3K4me3, and H3K27me3 signals at the range of the center of the Aged HSC open DARs ± 3.0 kb in aged HSCs. Aged HSC open DARs were subdivided into promoters and enhancers (active, primed, and inactive enhancers) based on their location and histone modification profiles. The number of DARs are indicated in parentheses. Histone modification profiles of the regions in young HSCs corresponding to Aged HSC open DARs are depicted in the right panels. **b** Chromatin accessibility (normalized ATAC counts) around Aged HSC open DARs in young and aged HSCs before and after stimulation with cytokines. **c** H3K27ac levels around Aged HSC open DARs in aged HSCs before and after stimulation with cytokines. **d** Box plots showing normalized ATAC counts (CPM) at Aged HSC open DARs (corresponding 37 promoters and 188 active, 91 primed, and 112 inactive enhancers) in young (left panels) and aged (right panels) HSCs before and after stimulation with cytokines. Data derived from a single experiment are depicted. Boxes represent the 25–75 percentile ranges. Horizontal bars represent medians. The ends of vertical lines represent minimum or maximum values. Paired Student’s two-tailed *t* tests were performed to calculate *p* values. **e** H3K27ac levels at Aged HSC open DARs (corresponding 37 promoters and 188 active, 91 primed, and 112 inactive enhancers) in aged HSCs before and after stimulation with cytokines. Data derived from a single experiment are depicted. ns, not significant. Paired Student’s one-tailed *t* tests were performed to calculate *p* values. Boxes represent the 25–75 percentile ranges. Horizontal bars represent medians. The ends of vertical lines represent minimum or maximum values. Source data are provided as a Source Data file.

We next performed profiling of H3K4me1, H3K27ac, H3K4me3, and H3K27me3 in young and aged HSCs. Most Aged HSC open DARs were located in non-promoter regions. We subdivided them into active (H3K4me1+/H3K27ac+), primed (H3K4me1+/H3K27ac−), and inactive (H3K4me1−/H3K27ac−) enhancers based on their histone modification status. No DARs were assigned to poised enhancers marked with H3K27me3 (Fig. 7a, Supplementary Data 7). Chromatin accessibility was increased in aged HSCs in all subgroups and was significantly enhanced at primed and inactive enhancers upon cytokine stimulation (Fig. 7b–d). H3K27ac levels were also significantly enhanced at primed enhancers upon cytokine stimulation (Fig. 7c–e). In contrast, chromatin accessibility and H3K27ac levels did not significantly change at active enhancers. These results indicate that Aged HSC open DARs comprised active enhancers associated with ongoing stresses and primed and inactive enhancers that can be readily activated upon re-exposure to their upstream stresses.

Our results suggest that a key difference between young and aged HSCs in chromatin accessibility is linked to stress exposure. We then examined whether the stress that mimics infection induces DARs that are retained as epigenetic memories in young HSCs. We challenged young mice with Lipopolysaccharide (LPS) and Polyinosinic:polycytidylic acid (polyI:C) 3 times a week for 4 weeks. To detect epigenetic memories in chromatin accessibility, we collected HSCs after 4 weeks of interval and performed ATAC-seq (Supplementary Fig. 11a). Unexpectedly, we found a very small number of DARs in treated HSCs 4 weeks after the challenge (Supplementary Fig. 11b). Accordingly, we did not detect significant changes at Aged HSC DARs in challenged young HSCs (Supplementary Fig. 11c, d). These results indicate that a short-term stress challenge is not sufficient to induce persistent chromatin accessibility changes or epigenetic memories in chromatin in young HSCs, or LPS and poly I:C cannot mimic the real microorganism infection in the induction of epigenetic memory in HSCs.

In this study, HSCs showed the least amount of expression changes, but more chromatin accessibility changes than progenitors. To understand this discrepancy, we evaluated chromatin accessibility around DEGs and found that the chromatin around DEGs was open and showed subtle changes between young and aged HSCs and progenitors (Supplementary Fig. 12), indicating that the expression changes of DEGs with aging do not necessarily accompany significant changes in chromatin accessibility compared with those at Aged HSC DARs detected in this study (Supplementary Fig. 12). In addition, nearly half of the open DARs in aged HSCs appeared to represent primed and inactive enhancers (Fig. 7a), which may also account for the weak correlation between DEG and DARs in aged HSCs. Thus, the weak correlation between DEGs and DARs in HSCs may represent the complex transcriptional and epigenetic regulation in HSCs.

## Discussion

In the present study, we comprehensively profiled the transcriptome and chromatin accessibility in aged HSCs. Transcriptome analysis revealed various differentially expressed genes between young and aged cells in each HSPC fraction, which were much more frequent in progenitors than in HSCs. Conversely, differences in chromatin accessibility tended to decrease during differentiation. We previously reported that switching the aged niche to a young niche largely restored the transcriptional profile of aged HSCs but not their DNA methylation profile^9^. It was also shown that DNA methylation and chromatin accessibility, but not the transcriptional state, in HSCs were relatively stable under various stress conditions, including different niches, suggesting that the epigenome and chromatin structure are responsible for the cell-autonomous behavior of HSCs^36^. These findings indicate that the integrated analysis of the epigenome and transcriptome provides more profound insight into HSC functions than the transcriptome alone.

Alterations in chromatin accessibility preferentially occurred in HSCs, the cells at the top of the hematopoietic hierarchy. The age-associated epigenetic changes in HSCs were considerably retained in MPP1, but gradually resolved and lost in downstream progenitor cells during differentiation. Thus, many of the stresses that target HSCs are thought to induce epigenetic changes preferentially in HSCs, which are not inherited by downstream progenitor cells. Binding motifs of the STAT, ATF, and CNC family transcription factors were significantly enriched in DARs in aged HSCs. These transcription factors are activated in response to external signals such as cytokine signals, inflammation, and oxidative stress, respectively^37–39^. The JAK-STAT pathway is activated by cytokine signals such as thrombopoietin, which promotes self-renewal of HSCs, but also IL-6 and interferons, inflammatory cytokines that affect HSC function. The inflammatory cytokine IL-1β also indirectly activates the JAK-STAT pathway. Among the ATF family transcription factors, Atf2, which forms a homodimer as well as a heterodimer with c-Jun, is activated by SAPKs (p38 and JNK) in response to various stresses, including inflammatory cytokines (IL-1β, IL-6), environmental stresses, and DNA damage^39^. The CNC family includes Nrf2, a master regulator of the antioxidant response, which is essential for maintaining HSCs and their stress-induced regenerative response^40^. Enrichment of these motifs may imply ongoing and/or history of exposure to such stresses and suggests that recurrent exposure to various stress signals induces changes in chromatin accessibility in HSCs.

There is accumulating evidence that supports the inheritance of epigenetic memory induced by various stresses. In particular, immunological memory has been well characterized^41,42^. For example, myeloid cells can show increased responsiveness upon subsequent stimulation with the same or a different stimulus after the initial challenge. This innate immune memory has been termed “trained immunity” and is involved in infections, vaccination, and inflammatory diseases. Trained immunity is mainly based on the epigenetic and metabolic reprogramming of cells^41,43^. Notably, it was shown that C/EBPβ-dependent epigenetic memory induces trained immunity in HSCs upon LPS challenge. Short-term LPS challenge can induce C/EBPβ-dependent chromatin accessibility, resulting in HSC-trained immunity during secondary infection^43^. Corresponding to these findings, a significant part of the genes linked to open DARs showed higher levels of basal expression augmented transcriptional responses upon cytokine stimulation in aged HSCs. Of interest, Aged HSC open DARs comprised not only active enhancers associated with ongoing stresses, but also primed and inactive enhancers that can be activated upon re-exposure to their upstream stresses. These results indicate that aged HSC-specific behaviors are partly scripted in the differentially accessible regions. Importantly, an excess of or sustained responses to external signals sometimes deteriorate hematopoietic homeostasis and impair HSC function, resulting in the promotion of HSC aging. Therefore, the unique behaviors of aged HSCs could be partly attributed to DAR-linked dysregulated stress responses of aged HSCs.

Many epigenomic alterations may also be merely passenger events with little biological meaning. Multiple studies have shown that most of the aberrant DNA methylation events in aged cells and cancers are passenger events, while only some methylation events are driver methylation events^44,45^. Likewise, the epigenomic alterations observed here may also include passenger events in HSC aging. It is challenging to distinguish epigenomic alterations that are crucial for stem cell aging from those that are merely passengers. Further investigation of the physiological and pathological roles of the epigenetic alterations profiled here would decipher the epigenetic mechanisms that contribute to the unique phenotypes of aged HSCs.

In summary, we performed an integrated analysis of the transcriptome and epigenome in aged HSCs and found an array of changes in chromatin accessibility and epigenome which transcriptome analysis could not detect. Our data provide key insights into aging research in hematopoiesis and will also serve as a reference for future studies.

## Methods

### Ethics approval

All experiments using mice were performed in accordance with our institutional guidelines for the use of laboratory animals and approved by the Review Board for Animal Experiments of Chiba University (approval ID: 30–56) and IMSUT (approval ID: PS18–02).

### Mice

Ten-week-old female C57BL/6 mice (B6-CD45.2) were purchased from Japan SLC (Japan) and bred for 16–20 months in the animal experiment facilities of Chiba University and The Institute of Medical Science, The University of Tokyo (IMSUT). Housing conditions were temperature 22 ± 2 °C, humidity 55 ± 5%, light/dark cycle 12 h/12 h (8 a.m.–20 p.m. light).

### Purification of HSCs and flow cytometry

BM cells were isolated by crashing bones from the backbone, pelvis, femurs, and tibiae. Flow cytometric analyses and cell sorting were performed using monoclonal antibodies recognizing the following antigens: CD45.2 (104 1:200 dilution), Gr-1 (RB6-8C5 1:200 dilution), CD11b/Mac-1 (M1/70 1:200 dilution), Ter-119 (TER-119 1:200 dilution), CD4 (GK1.5 1:200 dilution), CD8α (53–6.7 1:200 dilution), B220 (RA3-6B2 1:200 dilution), CD127/IL-7Rα (SB/199 or A7R34 1:100 dilution), CD117/c-Kit (2B8 1:200 dilution), Sca-1 (D7 1:200 dilution), CD135/Flk2 (A2F10 1:200 dilution), CD34 (RAM34 1:50 dilution), CD150 (TC15-12F12.2 1:100 dilution), CD48 (HM48-1 1:200 dilution), and CD16/32 [FcγRII-III (93 1:100 dilution)]. These antibodies were purchased from BD BioSciences, eBioScience, BioLegend, TOMBO, or R&D Systems. Cells were incubated with a mixture of biotin-conjugated monoclonal antibodies against lineage (Lin) markers including Gr-1, Ter-119, B220, CD4, CD8, and IL-7Rα (SB/199) (anti-IL-7Rα was removed from the mixture for CLP staining). Cells were stained further with fluorochrome-conjugated streptavidin and a combination of mAbs as follows: HSCs and MPPs; PerCP-5.5-Streptavidin, fluorescein isothiocyanate (FITC)-anti-CD34, phycoerythrin (PE)-anti-CD150, PE-Cy7-anti-Sca-1, Allophycocyanin (APC)-anti-c-Kit, APC-Cy7-anti-CD48, and Brilliant violet 421-anti-Flt3. GMPs and MEPs: APC-Cy7-streptavidin, FITC-anti CD34, PE-anti-FcγR, PE-Cy7-anti-Sca-1 and APC-anti-c-Kit. CLPs: PerCP-5.5-streptavidin, PE-anti-IL-7Rα (A7R34), PE-Cy7-anti-Sca-1, APC-anti-c-Kit, and Brilliant violet 421-anti-Flt3. Dead cells were removed by staining with 0.5 μg/mL propidium iodide (Sigma-Aldrich). All flow cytometric analyses and cell sorting were performed on FACS Aria III, FACS Canto II, or FACSCelesta (BD). Gating of young and aged HSPCs are indicated in Supplementary Fig. 13.

### Cell-cycle analysis

5-ethynyl-2′-deoxyuridine (EdU) (50 mg/kg) was injected intraperitoneally into mice 1 h before analysis. Cell-cycle status was determined by detecting incorporated EdU using a Click-iT^®^ EdU Flow Cytometry Assay Kit Alexa Fluor™ 647 (Thermo Fisher Scientific). DAPI at a concentration of 0.1 μg/ml was used to stain DNA content. Cells were incubated with a mixture of biotin-conjugated monoclonal antibodies against lineage markers including Gr-1, Ter-119, B220, CD4, CD8, and IL-7Rα (SB/199). Cells were stained further with fluorochrome-conjugated streptavidin and a combination of mAbs as follows: HSCs and MPPs; PerCP-Cy5.5-Streptavidin, FITC-anti-CD34, PE/Cy7-anti-Sca-1, APC/Cy7-anti-c-Kit, and PE-anti-Flt3. GMPs and MEPs: FITC-anti CD34, PE-anti-FcγR, PerCP-Cy5.5-Streptavidin, PE/Cy7-anti-Sca-1, and APC/Cy7-anti-c-Kit. CLPs: PerCP-Cy5.5-streptavidin, PE-anti-IL-7Rα (A7R34), PE/Cy7-anti-Sca-1, and APC/Cy7-anti-c-Kit.

### Stimulation of HSCs with cytokines

Young and aged HSCs were stimulated with a mixture of cytokines (SCF, TPO, GM-CSF, IL-1, IL-6, and IL-11, 10 ng/ml each) (BioLegend) in S-Clone SF-O3 (Sanko Junyaku) supplemented with 10% FBS, 50 μM 2-ME, and 1% GPS.

### RNA-seq and data processing

Total RNA was extracted using the RNeasy Plus Micro Kit (Qiagen) and subjected to reverse transcription and amplification using a SMARTer Ultra Low Input RNA Kit for Sequencing (Clontech). After sonication using an ultrasonicator (Covaris), the libraries for RNA-seq were generated from fragmented DNA using eight cycles of amplification by a NEB-Next Ultra DNA Library Prep Kit (New England BioLabs). After the libraries were quantified using a Bioanalyzer (Agilent), sequencing was performed using HiSeq2500 (Illumina) with a single-read sequencing length of 60 bp. TopHat2 (version 2.0.13; with default parameters) and Bowtie2 (version 2.1.0) were used for alignment to the reference mouse genome (mm10 from the University of California, Santa Cruz Genome Browser;) using the annotation data from iGenomes (Illumina). Normalization and removal of batch effects of count value and significant expression differences were detected using DESeq2 (version 2.2.1)^46^ with raw counts generated from StringTie. DEGs were defined using the cutoff of adjusted *p* values (*q* < 0.01). We used DAVID6.8 for gene ontology (GO) analysis. Normalized counts from DESeq2 were z-score scaled, and then subjected to *t*-SNE and expression heatmap. The value of Reads per kilobase of exon units per million mapped reads (RPKM) was calculated by StringTie (version 1.3.4). Motif analysis of promoters of DEGs was performed using Homer (findMotifs.pl, using the default parameters). The super-computing resources were provided by the Human Genome Center, the Institute of Medical Science, the University of Tokyo (http://sc.hgc.jp/shirokane.html).

### ATAC-seq and data processing

Regarding the ATAC sequence, a transposase reaction was performed using nuclei prepared from freshly sorted 20,000 cells of each fraction. Libraries were generated using a NEB-Next Ultra DNA Library Prep Kit (New England BioLabs, Beverly, MA, USA). Library DNA was size-selected (240–360 bps) using BluePippin (Sage Science, Beverly, MA, USA). Sequencing was performed using HiSeq1500 (Illumina) with a single-read sequencing length of 60 bp. Sequences were aligned to mouse genome sequences (mm10) using Bowtie2 (default setting). Mapped reads were subsampled using samtools to make the numbers of reads in all samples the same. Total read counts in each sample were subsampled to match the smallest one and standardized in library-size as count per million (CPM). In addition, CPM were also corrected in the step of calculation of differentially accessible regions by DESeq2 using the DESeq2 internal method called as the median-of-ratios method, which is similar to the trimmed mean of M values method (TMM)^46^. Macs2 (version 2.2.6) was used to call peaks using nomodel, a narrow peak option. Using a q-value cutoff of 0.001, accessible peaks were detected in each sample The catalog of all peaks called in any samples was produced by merging all called peaks that overlapped by, at least, one base pair using the Bedtools merge function. As a result, a total 102,983 peaks were detected and used as a map file for downstream processing. The Bedtools map function was used to count the reads at each region in the catalog using bed files of each sample. Read count matrix of each sample was used for detection of differentially accessible peaks (DARs) by using DESeq2. For the heatmap, normalized read counts obtained using DESeq2 were z-score-scaled and plotted. For motif analysis, findMotifsGenome.pl of Homer was used with the -size200-mask option. For the annotation of peaks, annotatePeaks.pl of Homer was used with the default settings. For visualization, RPM values of the sequenced reads were calculated for every 200-base pair bin with a shifting size of 100 base pairs by using bed tools, and then converted to a bigWig file using the wigToBigWig tool.

### scATAC-seq and data processing

Young and aged HSCs (10,000 cells) were collected for single cell ATAC-seq. Nuclei were isolated and libraries were prepared according to Chromium Next GEM Single Cell ATAC Reagent Kits v1.1 (10x Genomics). Raw data files (Base call files) were demultiplexed into fastq files using Cell-Ranger ATAC—2.0.0 with mkfastq command. Then, “cellranger-atac count” command was used for Read alignment, barcode counting and peak calling with reference “refdata-cellranger-arc-mm10-2020-A-2.0.0”, generating data of peaks, fragments, barcodes, and count matrix. Subsequent analyses were performed using Signac 1.4.0. For the integration of young and aged datasets, we used the GenomicRanges package to create combined peak regions. Quality control for each single cell were conducted based on these criteria (peak_region_fragments > 3000, peak_region_fragments < 20000, pct_reads_in_peaks > 15, blacklist_ratio < 0.05, nucleosome_signal < 4, and TSS.enrichment > 2). After quality control filtering, 5002 young and 3520 aged HSCs were used for further analysis. The feature plots of accessibility of Aged HSC open and closed regions were obtained using the function of “AddModuleScore”.

### CUT&TAG of histone modifications

Young and aged HSCs (60,000 cells) were collected as inputs. Libraries were prepared using CUT&Tag-IT™ Assay Kit (Active motif) according to manufacturer’s protocol. Anti-H3K27ac (#8173, Cell signaling) and anti-H3K4me1 (#39498, Active motif) were used for reaction. Sequencing was performed using HiSeq1500 (Illumina) with a single-read sequencing length of 60 bp. Sequences were aligned to mouse genome sequences (mm10) using Bowtie2 (default setting). For the peak calling and data normalization, Macs2 and DEseq2 were utilized in the same way as described in the section on ATAC-seq. BAM files are converted to bigwig format using deeptools with the command “bamCoverage -binSize 20 -normalizeUsing CPM -smoothLength 60”. Heatmap was created using deeptools “computeMatrix” and “plotHeatmap” functions. CPM were calculated using Bedtools. To separate the promoter and non-promoter regions, we used the “annotatePeaks.pl” function of the Homer program and defined a promoter as one that was annotated as a promoter-TSS in the default setting. For the definition of active, primed, and inactive enhancers, we assessed overlap of ATAC peaks, H3K4me1 peaks, and H3K27ac peaks generated from MACS2. To detect even the smallest of peaks of H3K4me1 and H3K27ac, we loosened the p value criterion to 0.01, and performed peak calls of histone modifications. If the peak overlapped with both H3K4me1 and H3K27ac peak, it was considered active; if it overlapped with H3K4me1 and not with H3K27ac, it was considered primed; and if it did not overlap with H3K4me1, it was considered inactive.

### ChIP-seq and data processing

A ChIP analysis was performed as described previously^47,48^. Cells were cross-linked with 0.5% formaldehyde at 37 °C for 2 min, washed with PBS containing 2% FBS, lysed with ChIP buffer (10 mM Tris-HCl pH 8.0, 200 mM NaCl, 1 mM CaCl2, 0.5% NP40, and protease inhibitor cocktail), and sonicated for 5 s × 3 times (90 s on ice) using a Bioruptor (Cosmo Bio). Cells were digested with micrococcal nuclease (MNase) (NEB) at 37 °C for 40 min and treated with 10 mM EDTA to stop the reaction. After the addition of an equal volume of RIPA buffer (50 mM Tris-HCl pH 8.0, 150 mM NaCl, 2 mM EDTA, 1% NP40, 0.5% sodium deoxycholate, and 0.1% SDS), cells were resonicated for 5 s × 10 times (300 s on ice) using the Bioruptor. After centrifugation, the soluble chromatin fraction was immunoprecipitated at 4 °C overnight with Dynabeads M-280 Sheep anti-Rabbit IgG conjugated with an anti-H3K27me3 (07-449, Millipore), or anti-H3K4me3 (07-473, Millipore) antibody. Immunoprecipitates were washed with high salt ChIP buffer (500 mM NaCl) four times and TE buffer (10 mM Tris-HCl pH 8.0 and 1 mM EDTA) twice. Bound chromatin and 25 μl of input DNA were suspended in 47.5 and 22.5 μl of elution buffer (50 mM Tris-HCl pH 8.0, 10 mM EDTA, 1% SDS, and 250 mM NaCl), respectively, and vortexed. After the addition of 5 μl of 5 M NaCl, the solutions were incubated at 65 °C for 4 hr and then treated with 25 μg/ml RNase A (Sigma-65 Aldrich) at 37 °C for 30 min and 0.1 mg/ml proteinase K (Roche) at 50 °C for 1 h to reverse formaldehyde crosslinking. Eluted DNA was purified with a MinElutePCR Purification Kit (Qiagen). DNA libraries were prepared from input and ChIP DNA samples using a ThruPLEX DNA-seq Kit (Rubicon Genomics) according to the manufacturer’s instructions. DNA libraries were quantified using high sensitivity Chip on the Bioanalyzer (Agilent) and sequencing was performed using HiSeq1500 (Illumina) with a single-read sequencing length of 60 bp. Sequences were aligned to mouse genome sequences (mm10) with Bowtie2 (default setting). BAM files are converted to bigwig format using deeptools with the command “bamCoverage -binSize 20 -normalizeUsing CPM -smoothLength 60”. Heatmap was created using deeptools “computeMatrix” and “plotHeatmap” functions.

### WGBS data processing

We used deposited data of DRA009270. Data processing was performed as described previously.^9^ In summary, WGBS data were mapped to the mouse genome (mm10) using Bismark software (Babraham Institute, Cambridge, UK). Methylation scores were calculated as the number of unconverted reads divided by the number of total reads at ATAC peak regions.

### Statistical analysis

Statistical tests were performed using GraphPad Prism version 8. The statistical test used for each analysis was indicated in figure legends

### Reporting summary

Further information on research design is available in the Nature Research Reporting Summary linked to this article.

## Supplementary information

Supplementary Information

Description of Additional Supplementary Files

Supplementary Data 1

Supplementary Data 2

Supplementary Data 3

Supplementary Data 4

Supplementary Data 5

Supplementary Data 6

Supplementary Data 7

Reporting Summary

## Data Availability

The NGS data generated in this study have been deposited in the NCBI Gene Expression Omnibus as SuperSeries under accession code GSE162662 composed of the following SubSeries: GSE162551 (ATAC-seq), GSE162607 (RNA-seq), and GSE169206 (RNA-seq after cytokine stimulation), GSE190422 (RNA-seq of MPPs after cytokine stimulation), GSE190419 (ATAC-seq in the cytokine and LPS/polyI:C challenge experiments), GSE190420 (CUT&TAG-seq), GSE162570 (ChIP-seq), and GSE190424 (single cell ATAC-seq). Source data are provided with this paper.

## Source data

Source Data

## Code availability

All codes and scripts used for the analyses are stored in Github [https://github.com/Itokawa-Naoki/Aging_HSC] and deposited to repository with the following 10.5281/zenodo.6336320 [10.5281/zenodo.6336320].
