# Supplementary material for: Epigenetic traits inscribed in chromatin accessibility in aged hematopoietic stem cells
Source: Nat Commun. 2022 May 16;13:2691. doi: 10.1038/s41467-022-30440-2 (PMC9110722; doi:10.1038/s41467-022-30440-2)
Supplement: Supplementary file 1 — Supplementary Information [file 41467_2022_30440_MOESM1_ESM.pdf]

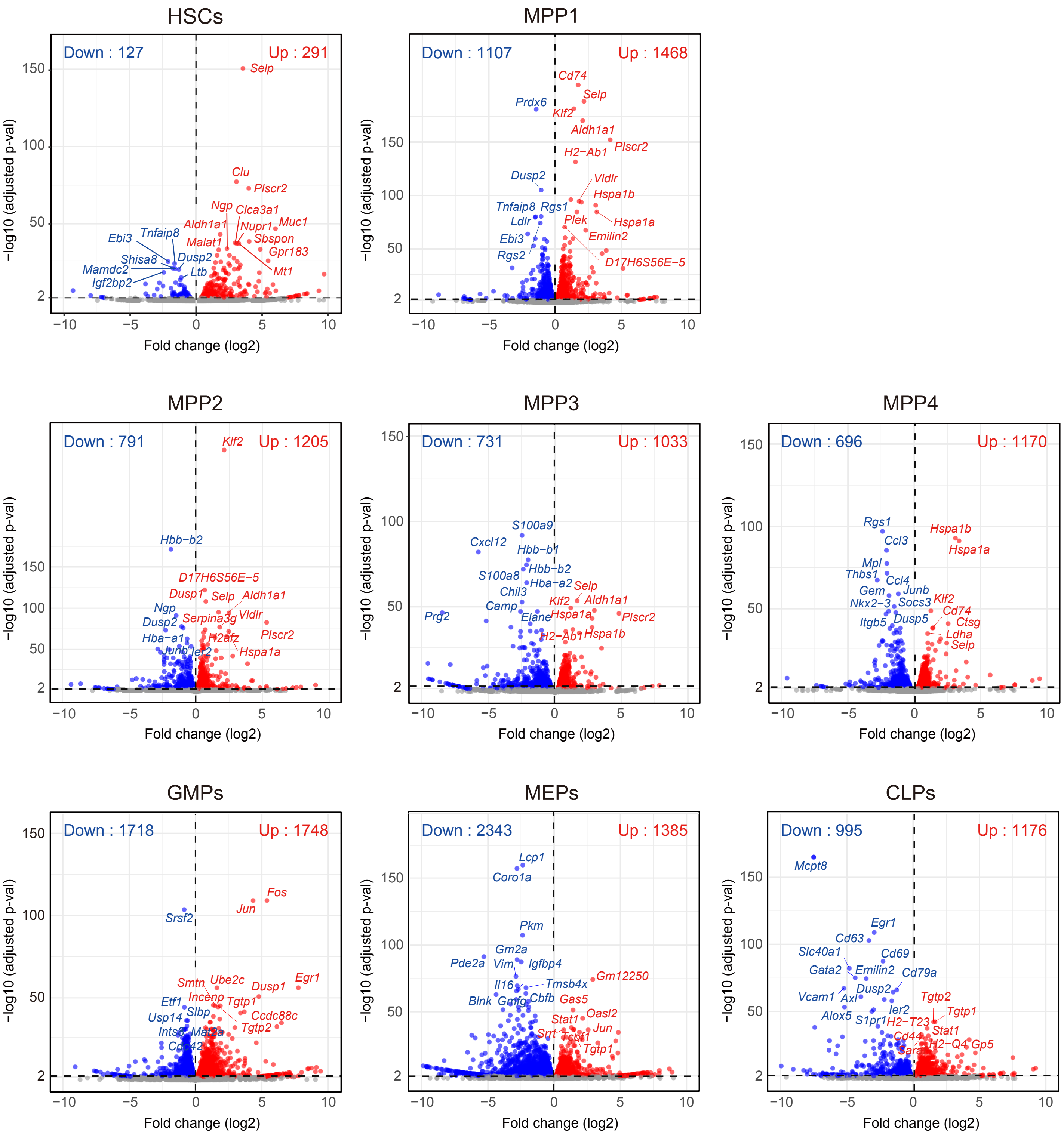

Supplementary Figure 1. Differentially expressed genes in age HSPCs
Volcano plot showing the differentially expressed genes (DEGs) between young and aged counterparts in each HSPC fraction. The vertical axis (y-axis) displays adjusted p-values, and the horizontal axis (x-axis) displays the log2 fold change values. The red and blue dots represent the up (UP) and down (DOWN) DEGs with aging. A cutoff $q < 0.01$ was used to define the DEGs. P-values were calculated by Generalized linear model. Multiple comparisons were corrected as q-values by Benjamini-Hochberg method.

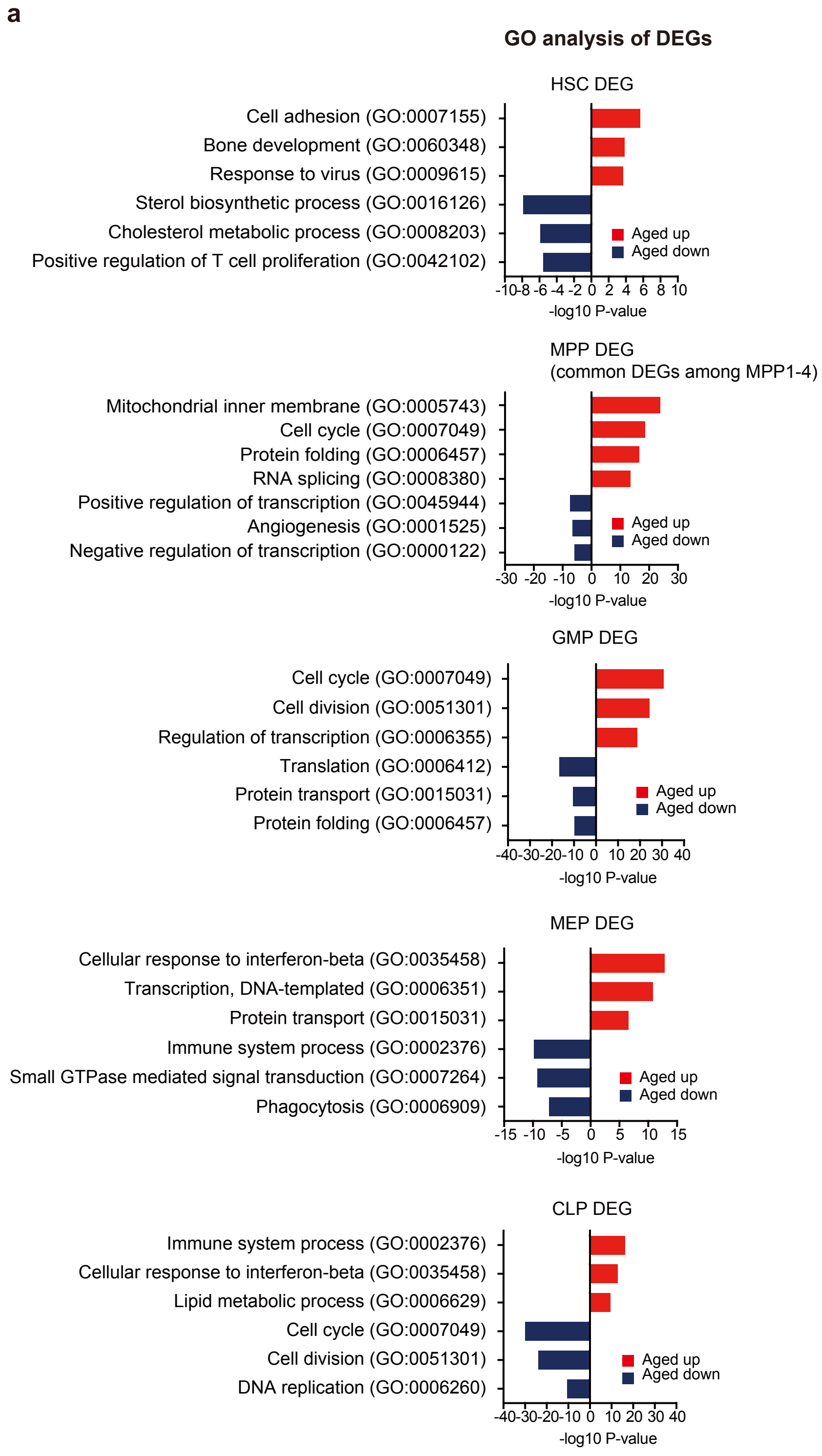

b

Promoter Motif analysis of DEGs

HSC DEG

No significant motif | No significant motif

MPP DEG
(common DEGs among MPP1-4)

Aged up	*p*-value
NFY (CCAAT)	10^{-13}
E2F7 (E2F)	10^{-12}
E2F4 (E2F)	10^{-12}
Elk4	10^{-7}

No significant motif

GMP DEG

Aged up	*p*-value	Aged down	*p*-value
YY1 (Zf)	10^{-13}	Elk1 (ETS)	10^{-23}
ISRE (IRF)	10^{-6}	Elk4 (ETS)	10^{-22}
NFY (CCAAT)	10^{-5}	ELF1 (ETS)	10^{-21}
IRF1 (IRF)	10^{-4}	GABPA (ETS)	10^{-18}

MEP DEG

Aged up	*p*-value	Aged down	*p*-value
YY1 (Zf)	10^{-16}	SpiB (ETS)	10^{-11}
ISRE (IRF)	10^{-10}	PU.1 (ETS)	10^{-9}
Elk4 (ETS)	10^{-9}	Fli1 (ETS)	10^{-9}
ETS (ETS)	10^{-9}	GABPA (ETS)	10^{-7}

CLP DEG

Aged up	*p*-value	Aged down	*p*-value
ISRE (IRF)	10^{-17}	NFY (CCAAT)	10^{-11}
IRF2 (IRF)	10^{-15}	E2F1 (E2F)	10^{-8}
IRF1 (IRF)	10^{-13}	E2F4 (E2F)	10^{-7}
PU.1:IRF8	10^{-8}	E2F7 (E2F)	10^{-6}

Supplementary Figure 2. Gene Ontology and motif analyses of differentially expressed genes
(a) Gene Ontology (GO) analysis of DEGs. Significant biological process GO terms were selected. The horizontal axis indicates the -log10 p-values. Red and blue bars represent the enriched terms in aged up and down DEGs, respectively. P-values were culculated by Fisher's Exact test. No adjustments were made for multiple comparisons. (b) Motif analysis of promoters of DEGs in each fraction. Motif name and p-values are shown. Hypergeometric tests were performed to calculate p-values. No adjustments were made for multiple comparisons.

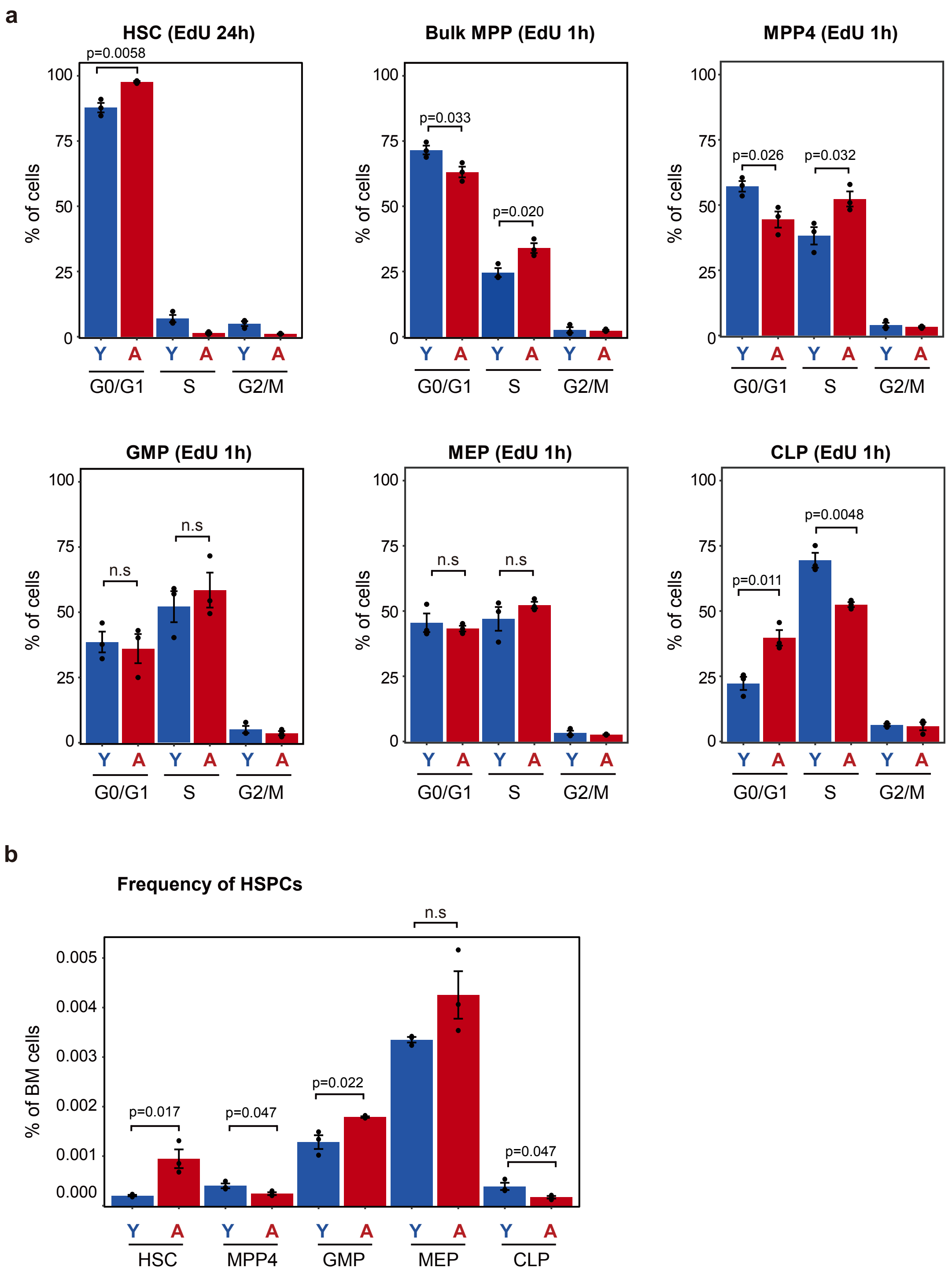

Supplementary Figure 3. Cell cycle status of young and aged HSPCs
(A) Cell cycle of young and aged HSPCs detected by EdU incorporation assays. EdU was injected into mice 24 hours and 1 hour before the analysis of HSCs and other progenitors, respectively. Data are shown as the mean ± SEM (n=3, biologically independent samples). ns, not significant. Unpaired two-tailed Student's *t*-tests was used to calculate p-values.
(B) Proportions of HSPCs in the BM of young and aged mice. Data are shown as the mean ± SEM (n=3, biologically independent samples). ns, not significant. Unpaired two-tailed Student's *t*-tests.

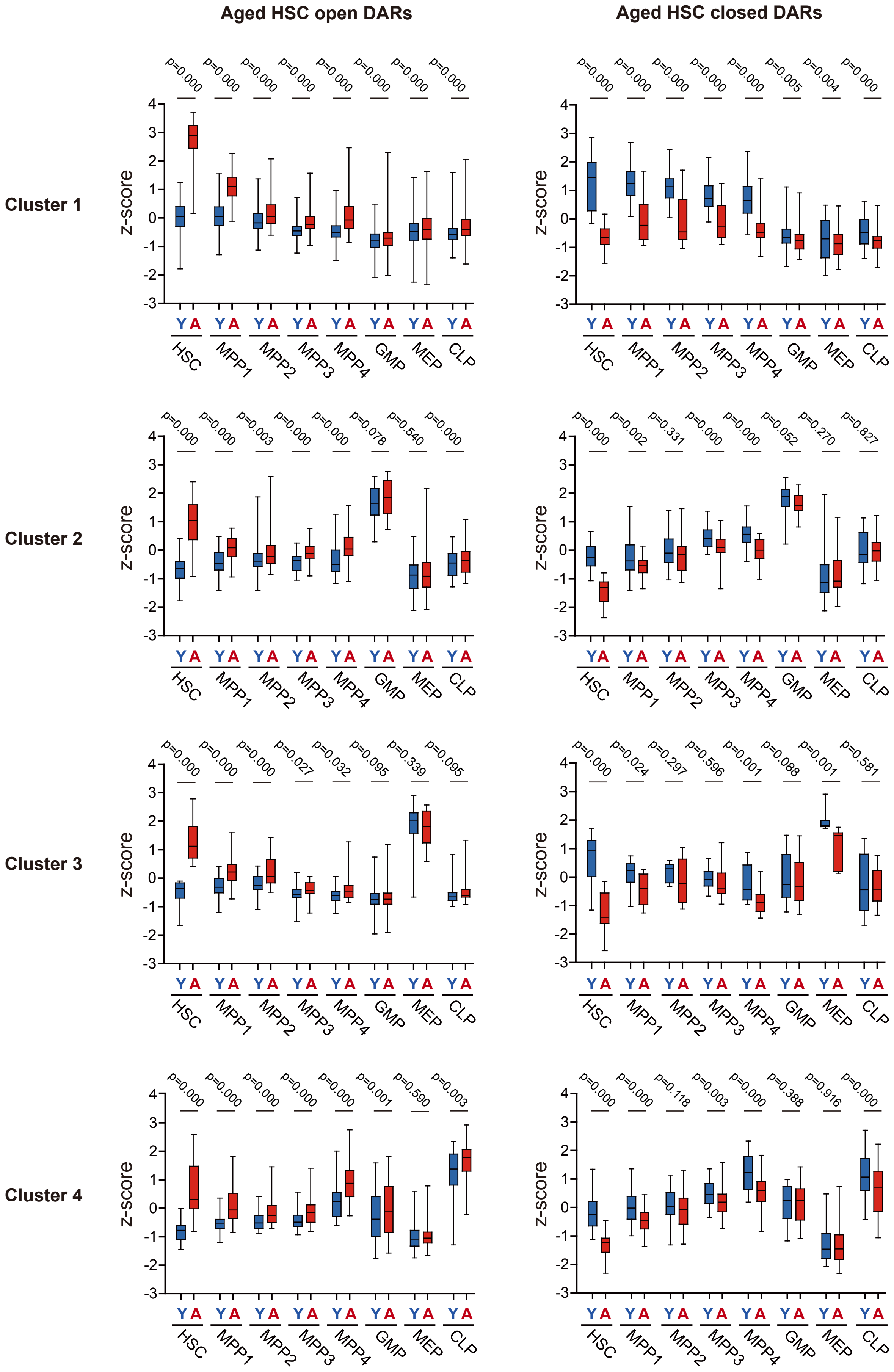

Supplementary Figure 4. Chromatin accessibility changes during differentiation

Box plot showing accessibility changes during differentiation (z-scores of DEseq2 normalized ATAC counts) in Aged HSC open (left panels) and closed (right panels) DARs. DARs are divided into clusters. The horizontal bars indicate the median values. Open DARs [Cluster 1 (329), Cluster 2 (38), Cluster 3 (26), and Cluster 4 (35)] and closed DARs [Cluster 1 (45), Cluster 2 (32), Cluster 3 (9), and Cluster 4 (33)] defined using 2 biologically independent samples were examined. Boxes represent the 25–75 percentile ranges. Horizontal bars represent medians. The ends of vertical lines represent minimum or maximum values.

Aged MPP1 open DARs

Motif	Name	P (-log10)	% in DAR	% in Y-MPP1 peaks	% in all peaks
	ISRE	11.6	11.7	1.2	1.5
	IRF1	6.0	11.7	2.2	3.5
	IRF2	5.9	10.2	2.1	2.7
	Atf1	5.4	20.5	8.3	9.5

Aged MPP1 close DARs

Motif	Name	P (-log10)	% in DAR	% in Y-MPP1 peaks	% in all peaks
	AMYB	16.0	46.0	15.7	14.5
	MYB	14.8	48.0	19.0	16.8
	BMYB	12.8	40.0	14.8	13.3
	PBX1	8.7	10.0	1.5	1.04

Aged MPP2 open DARs

Motif	Name	P (-log10)	% in DAR	% in Y-MPP2 peaks	% in all peaks
	IRF2	6.4	13.6	1.9	2.9
	ISRE	5.2	9.1	1.0	1.6
	Klf4	5.0	15.9	9.6	5.1
	PRDM1	4.1	15.9	4.4	6.2

Aged MPP2 close DARs

Motif	Name	P (-log10)	% in DAR	% in Y-MPP2 peaks	% in all peaks
	BMYB	9.2	32.2	14.8	15.9
	AMYB	8.1	32.2	15.5	16.9
	Fra1	7.9	22.2	10.2	9.7
	MYB	6.8	33.3	18.9	19.2

Aged MPP3 open DARs

Motif	Name	P (-log10)	% in DAR	% in Y-MPP3 peaks	% in all peaks
	ISRE	10.8	20.8	1.1	1.4
	IRF1	9.3	25.0	2.1	3.2
	IRF2	7.9	20.8	2.1	2.6
	GATA2	4.6	25.0	6.6	7.9
	PRDM1	4.2	20.8	4.6	6.7

Aged MPP3 close DARs

Motif	Name	P (-log10)	% in DAR	% in Y-MPP3 peaks	% in all peaks
	BMYB	8.2	35.5	14.8	14.1
	MYB	7.7	40.0	18.9	17.8
	AMYB	7.2	35.5	15.5	15.4
	PBX1	6.6	8.9	1.3	1.1
	RFX	6.5	11.1	2.2	1.8

Aged MPP4 open DARs

Motif	Name	P (-log10)	% in DAR	% in Y-MPP4 peaks	% in all peaks
	ISRE	15.1	11.7	1.3	1.6
	IRF2	14.6	14.8	2.2	2.8
	IRF1	10.5	13.8	2.4	3.5
	PU1:IRF8	9.5	17.0	3.5	5.7
	Bcl6	7.6	25.5	10.2	12.5
	PRDM1	7.2	15.9	4.9	6.3

Aged MPP4 close DARs

Motif	Name	P (-log10)	% in DAR	% in Y-MPP4 peaks	% in all peaks
	PBX1	8.4	7.1	1.3	1.0
	ZNF189	6.5	17.8	7.2	6.5

Aged GMP open DARs

Motif	Name	P (-log10)	% in DAR	% in Y-GMP peaks	% in all peaks
	Gata2	10.1	26.3	4.4	8.2
	Gata1	9.6	24.5	4.0	7.5
	ETS:RUNX	6.9	14.0	4.1	3.6

Aged GMP close DARs

Motif	Name	P (-log10)	% in DAR	% in Y-GMP peaks	% in all peaks
	RFX	11.5	23.8	1.8	1.4
	PBX1	9.6	19.0	1.2	1.1

Aged MEP close DARs

Motif	Name	P (-log10)	% in DAR	% in Y-MEP peaks	% in all peaks
	PBX1	8.4	27.2	1.7	1.1
	RFX	7.9	27.2	2.2	1.3

Aged CLP close DARs

Motif	Name	P (-log10)	% in DAR	% in Y-CLP peaks	% in all peaks
	RFX	6.0	17.6	2.2	1.6

Supplementary Figure 5. Motif enriched in open and closed DARs in aged progenitors.
Motifs and -log10 p-values are depicted. The enrichment of each motif in DARs in aged progenitors, the peaks in young progenitors, and the background peaks are indicated. All peaks, which includes 102,992 peaks from all fractions, were used as background. P-values were derived from Hypergeometric tests of motif analysis. No adjustments were made for multiple comparisons. Source data are provided as a Source Data file.

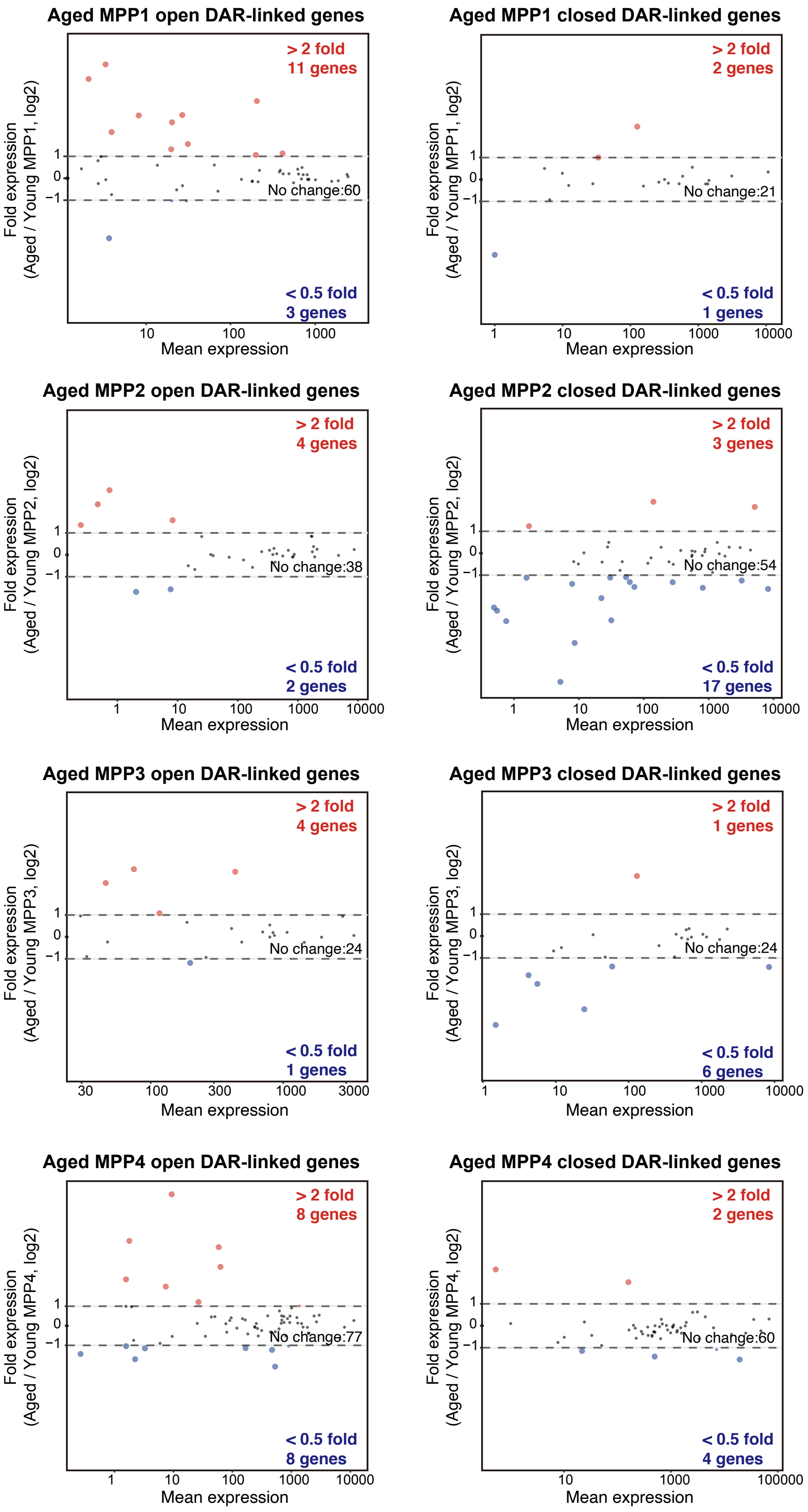

Supplementary Figure 6. MA plots showing expression of Aged progenitor DAR-linked genes. The pink and blue dots represent up- and down-regulated DAR-linked genes in aged MPPs greater than 2-fold relative to young MPPs, respectively.

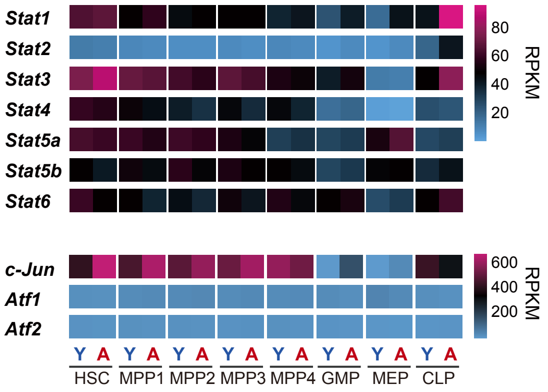

Supplementary Figure 7. Expression of transcription factor genes enriched in Aged HSC open DARs
Heatmap showing the expression values (RPKM) of STAT and ATF family transcription factor genes and *c-Jun*.

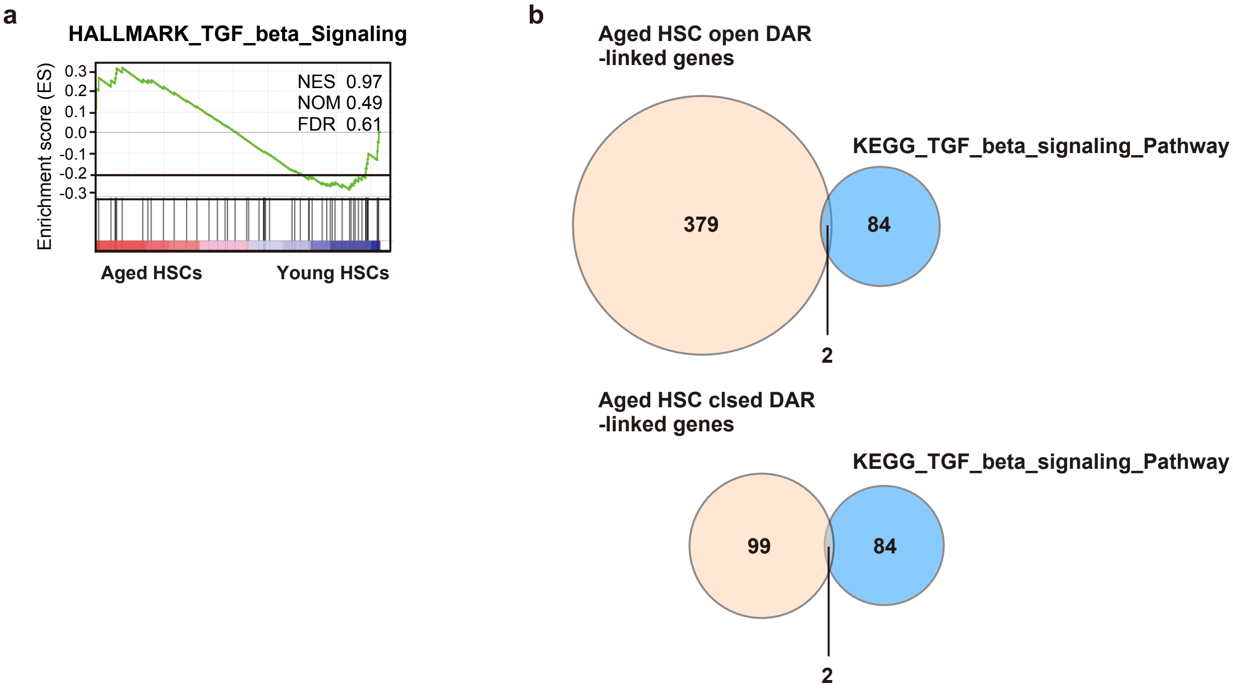

Supplementary Figure 8. TGF-β signaling in aged HSCs
(a) GSEA plot for HALLMARK_TGF_beta_Sigfnaling (M5896) gene set in aged HSCs compared with young HSCs. Normalized enrichment scores (NES), nominal p values (NOM), and false discovery rates (FDR) are indicated.
(b) Venn diagrams showing the overlap between Aged HSC open and closed DAR-linked genes with KEGG_ TGF_beta_Signaling_Pathway (M2642) gene set.

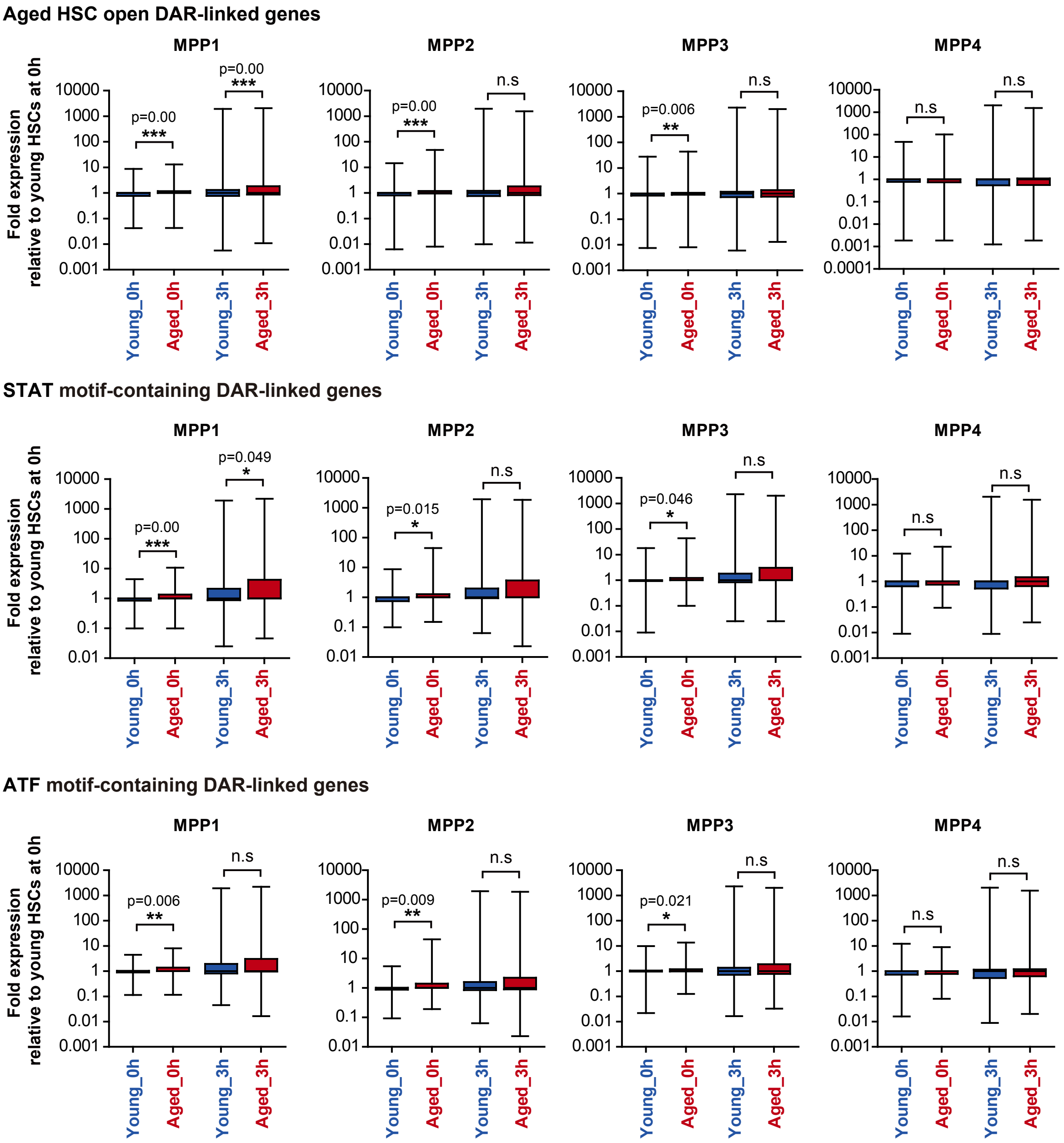

Supplementary Figure 9. Cytokine responsiveness of DAR-linked genes in progenitors

Fold expression of DAR-linked genes in young and aged MPPs after cytokine stimulation. Young and aged MPPs were stimulated with a mixture of cytokines (SCF, TPO, GM-CSF, IL-1, IL-6, and IL-11) in vitro and subjected to RNA-seq analysis at the indicated time points. Expression of DAR-linked genes is indicated as a fold expression relative to that in young HSCs (0 hour) using normalized read counts (DESeq2 normalized counts +1). DAR-linked genes (381), STAT motif-containing DAR -linked genes (103), and ATF motif-containing DAR-linked genes (116) were examined. Data derived from a single experiment are depicted. Boxes represent the 25–75 percentile ranges with the median of horizontal line. The ends of vertical lines represent minimum or maximum values. P-values were calculated by the paired one-tailed Student’s t-tests.

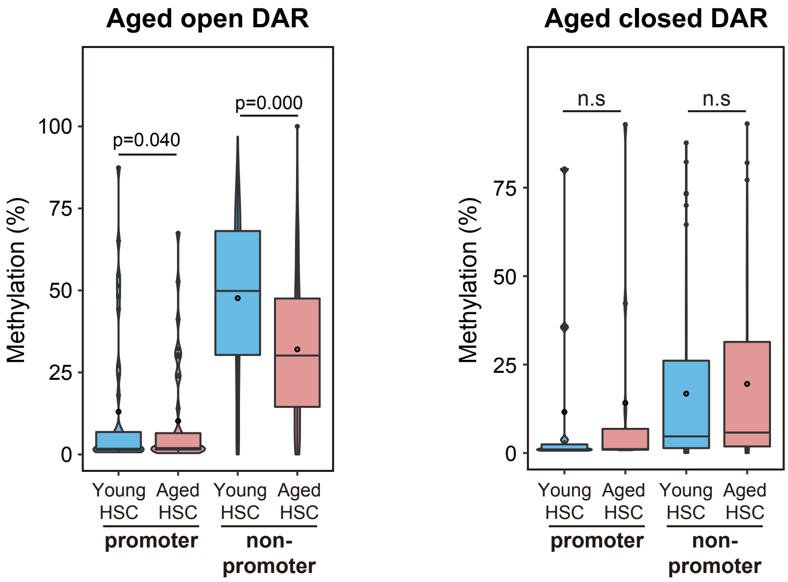

Supplementary Figure 10. DNA methylation status of Aged HSC DARs
Re-analyses of whole-genome bisulfite sequencing (WGBS) data sets of HSCs from 10-week-old and 20-month-old mice (Ref. 9). Box and violin plots showing the DNA methylation levels (%) of open and closed DARs in aged HSCs. Horizontal bars and circles indicate the median and mean values, respectively. Promoter open DARs (37), non-promoter open DARs (383), promoter closed DARs (11), and non-promoter closed DARs (100) were examined. Boxes represent the 25–75 percentile ranges with the median of horizontal lines. The ends of vertical lines represent minimum or maximum values. n.s., not significant. P-values were calculated by the paired two-tailed Student's *t*-tests.

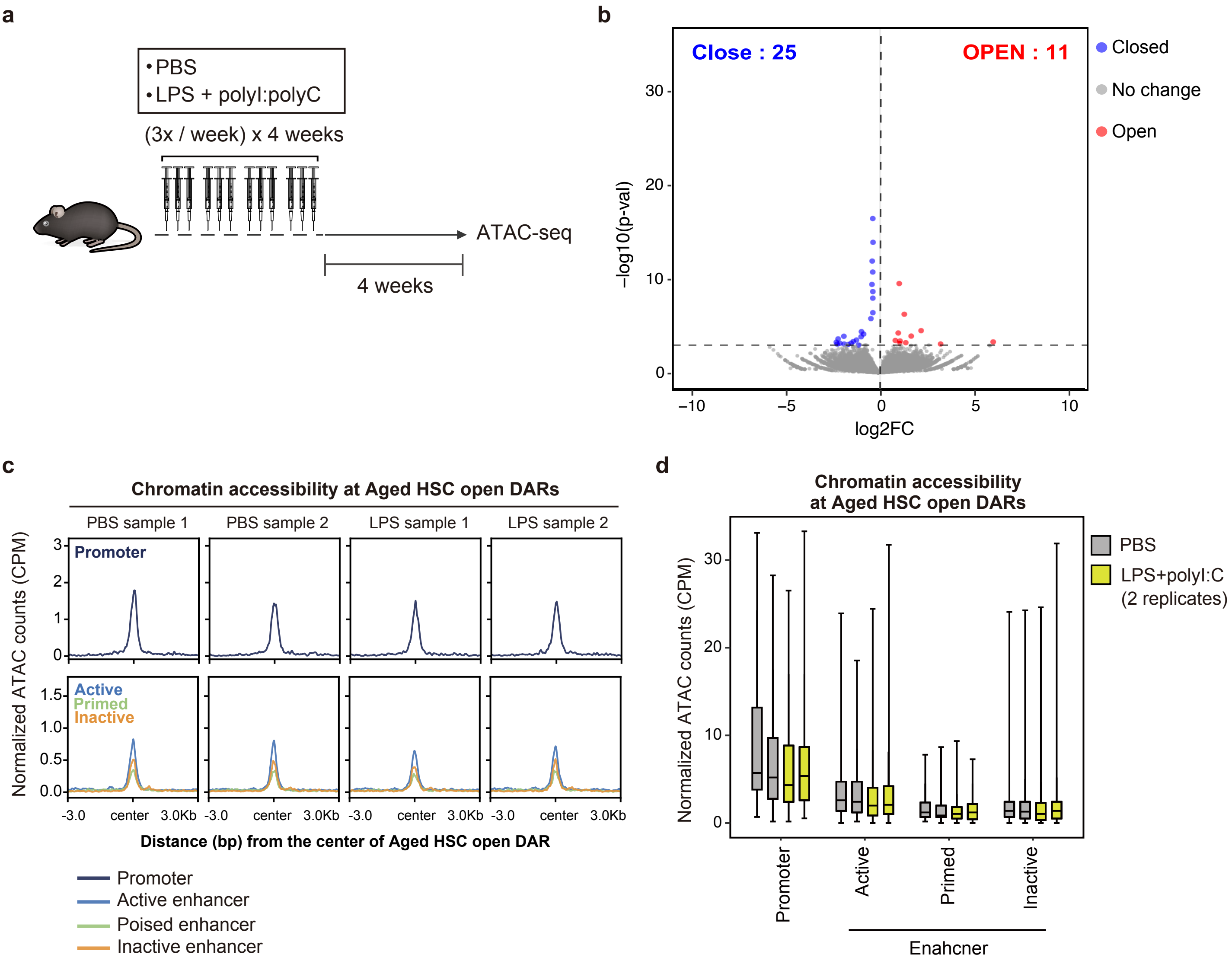

Supplementary Figure 11. Chromatin accessibility in young HSCs challenged with infection mimetic stress
(a) Experimental strategy. Young mice (10 weeks old) were challenged with LPS and polyI:polyC 3 times a week for 4 weeks. After 4 weeks of interval, we collected HSCs and performed ATAC-seq.
(b) Scatter plot showing chromatin accessibility changes in young HSCs 4 weeks after the challenge relative to PBS control HSCs. P-values were calculated by Generalized linear model. No adjustments were made for multiple comparisons. DARs were identified using z-scores of DEseq2 normalized ATAC counts of biological duplicates (n=2) and defined those $p < 0.001$.
(c) Normalized ATAC counts (count per million, CPM) around Aged HSC open DARs in PBS control and challenged young HSCs.
(d) Box plots showing normalized ATAC counts (CPM) at Aged HSC open DARs (corresponding to 37 promoters and 188 active, 91 primed, and 112 inactive enhancers in aged HSCs) in PBS control and challenged young HSCs. Boxes represent the 25–75 percentile ranges with the median of horizontal lines. The ends of vertical lines represent minimum or maximum values.

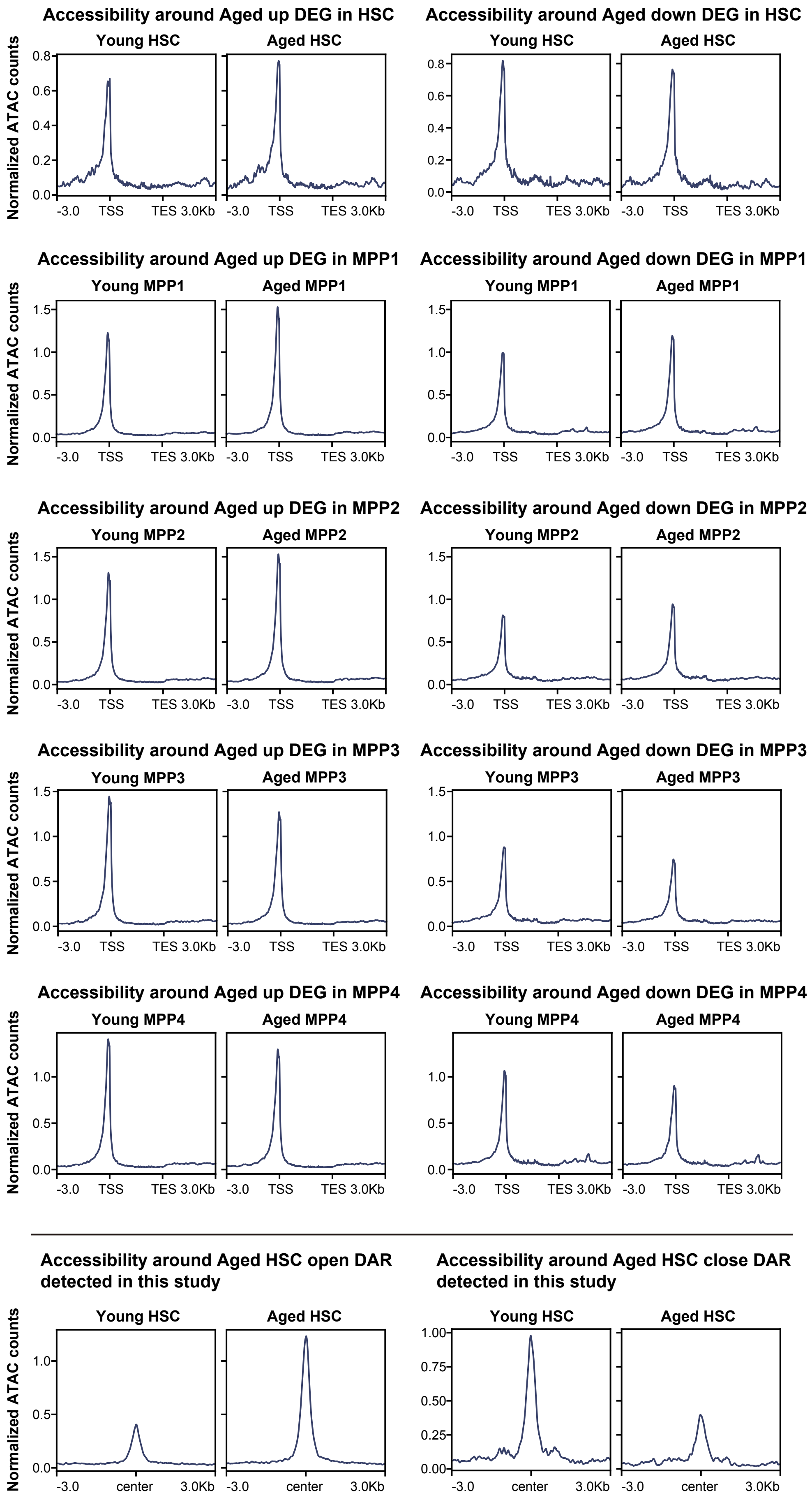

Supplementary Figure 12. Chromatin accessibility around DEGs

Normalized ATAC counts (count per million, CPM) around DEGs (up and down) in aged HSCs and MPPs. Normalized ATAC counts around Aged HSC DARs (open and closed) are depicted on the bottom.

Young HSPC Sorting

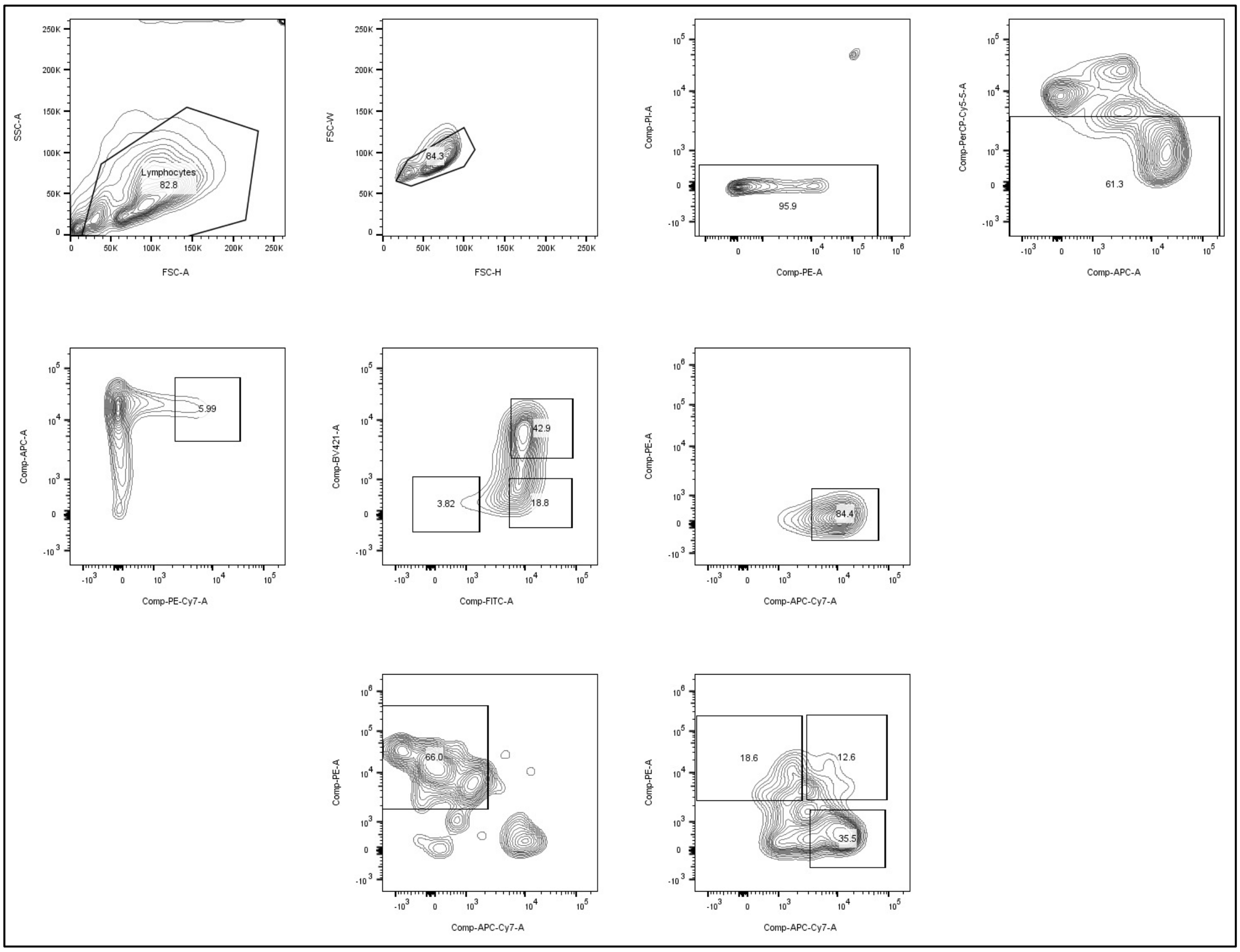

Aged HSPC Sorting

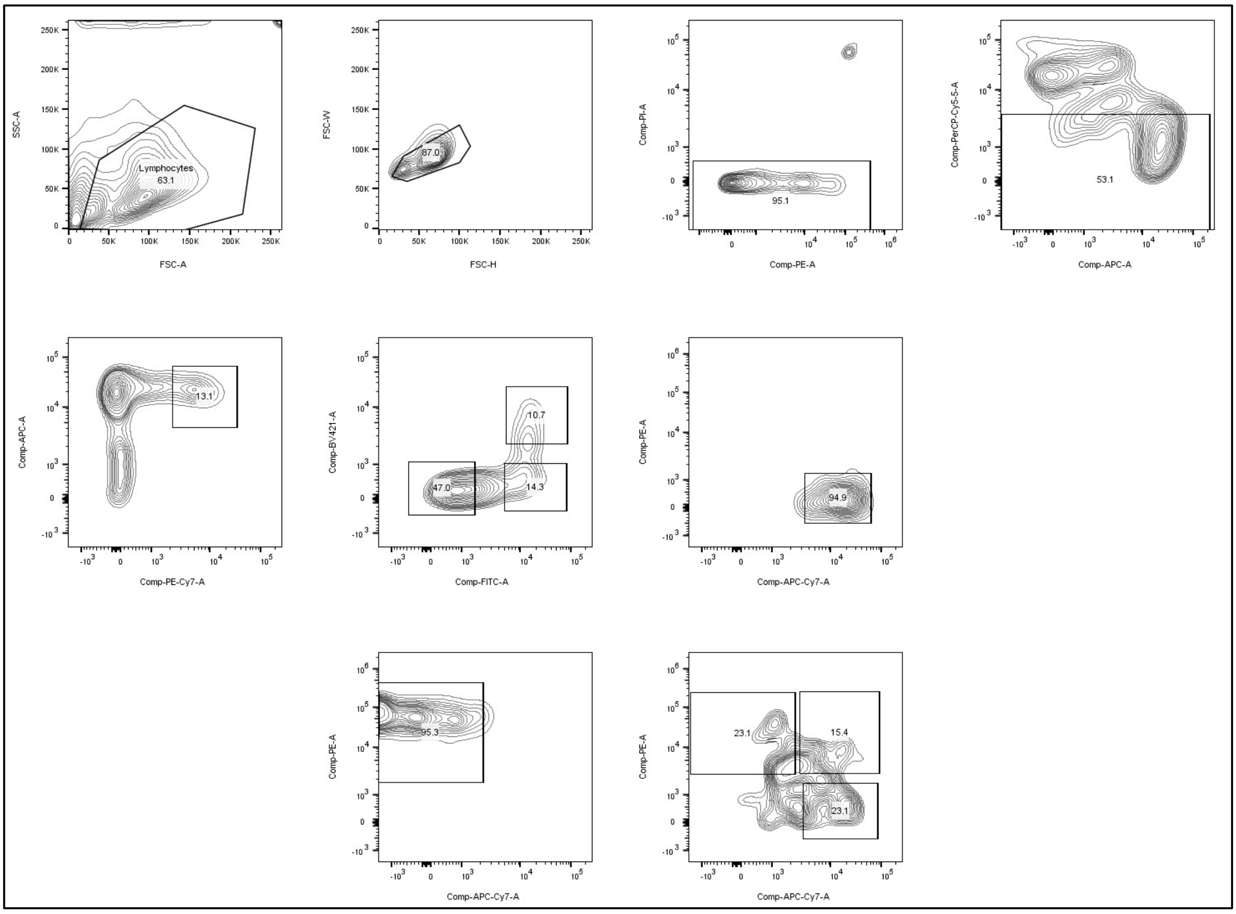

Supplementary Figure 13. FACS sorting strategy for hematopoietic stem and progenitor fractions (HSPCs)
