# Supplementary material for: Epigenetic traits inscribed in chromatin accessibility in aged hematopoietic stem cells
Source: Nat Commun. 2022 May 16;13:2691. doi: 10.1038/s41467-022-30440-2 (PMC9110722; doi:10.1038/s41467-022-30440-2)
Supplement: Supplementary file 2 — Description of Additional Supplementary Files [file 41467_2022_30440_MOESM2_ESM.docx]

**Description of Additional Supplementary Files**

**Supplementary Data 1.** Differentially expressed genes (DEGs) between young and aged HSPCs

**Supplementary Data 2.** GO analysis of aged down DEGs in HSPCs

**Supplementary Data 3.** Motifs enriched at promoters of aged up DEGs in progenitors

**Supplementary Data 4.** Motif analysis of ATAC peaks

**Supplementary Data 5.** Differentially accessible regions (DARs) between young and aged HSPCs and Homer motif analysis of aged open DARs in HSPCs

**Supplementary Data 6.** Expression of Aged HSC open DAR-linked genes in HSCs stimulated by cytokines

**Supplementary Data 7.** Annotation of Aged HSC open DARs based on H3K27ac and H3K4me1 datasets
