# Supplementary material for: Epigenetic traits inscribed in chromatin accessibility in aged hematopoietic stem cells
Source: Nat Commun. 2022 May 16;13:2691. doi: 10.1038/s41467-022-30440-2 (PMC9110722; doi:10.1038/s41467-022-30440-2)
Supplement: Supplementary file 10 — Reporting Summary [file 41467_2022_30440_MOESM10_ESM.pdf]

nature portfolio

Corresponding author(s): Atsushi Iwama

Last updated by author(s): Mar 16, 2022

Reporting Summary

Nature Portfolio wishes to improve the reproducibility of the work that we publish. This form provides structure for consistency and transparency in reporting. For further information on Nature Portfolio policies, see our Editorial Policies and the Editorial Policy Checklist.

Statistics

For all statistical analyses, confirm that the following items are present in the figure legend, table legend, main text, or Methods section.

n/a	Confirmed	
☐	☒	The exact sample size (n) for each experimental group/condition, given as a discrete number and unit of measurement
☐	☒	A statement on whether measurements were taken from distinct samples or whether the same sample was measured repeatedly
☐	☒	The statistical test(s) used AND whether they are one- or two-sided<br>*Only common tests should be described solely by name; describe more complex techniques in the Methods section.*
☒	☐	A description of all covariates tested
☐	☒	A description of any assumptions or corrections, such as tests of normality and adjustment for multiple comparisons
☐	☒	A full description of the statistical parameters including central tendency (e.g. means) or other basic estimates (e.g. regression coefficient) AND variation (e.g. standard deviation) or associated estimates of uncertainty (e.g. confidence intervals)
☐	☒	For null hypothesis testing, the test statistic (e.g. F, t, r) with confidence intervals, effect sizes, degrees of freedom and P value noted<br>*Give P values as exact values whenever suitable.*
☒	☐	For Bayesian analysis, information on the choice of priors and Markov chain Monte Carlo settings
☒	☐	For hierarchical and complex designs, identification of the appropriate level for tests and full reporting of outcomes
☒	☐	Estimates of effect sizes (e.g. Cohen's d, Pearson's r), indicating how they were calculated

Our web collection on statistics for biologists contains articles on many of the points above.

Software and code

Policy information about availability of computer code

Data collection	Detailed information about cells fractionation, library preparation, and next generation sequence are described in the method section.<br>In summary, FACSAria III, FACSCelesta (BD), SMARTer Ultra Low Input RNA Kit, NEBNext Ultra DNA Library Prep Kit, and HiSeq2500 were used.
Data analysis	All software were described in the method section and code and script are open in github (https://github.com/Itokawa-Naoki/Aging_HSC)<br>bowtie2(2.1.0), tophat2(2.0.13), stringtie(1.3.4), samtools(1.9), bedtools(2.27.1), Bismark(0.22.3), deeptools(3.3.1)<br>R(4.0.2), macs2(2.2.6), DESeq2(1.34.0), Homer(4.11), DAVID6.8, GraphPad Prism 9, wigToBigWig(4)

For manuscripts utilizing custom algorithms or software that are central to the research but not yet described in published literature, software must be made available to editors and reviewers. We strongly encourage code deposition in a community repository (e.g. GitHub). See the Nature Portfolio guidelines for submitting code & software for further information.

Data

Policy information about availability of data

All manuscripts must include a data availability statement. This statement should provide the following information, where applicable:

- Accession codes, unique identifiers, or web links for publicly available datasets
- A description of any restrictions on data availability
- For clinical datasets or third party data, please ensure that the statement adheres to our policy

NGS data have been deposited in the NCBI Gene Expression Omnibus as SuperSeries of GSE162662 [https://www.ncbi.nlm.nih.gov/geo/query/acc.cgi?acc=GSE162662] composed of the following SubSeries: GSE162551 (ATAC-seq), GSE162607 (RNA-seq), and GSE169206 (RNA-seq after cytokine stimulation), GSE190422 (RNA-seq of MPPs after cytokine stimulation), GSE190419 (ATAC-seq in the cytokine and LPS/polyI:C challenge experiments), GSE190420 (CUT&TAG-seq), GSE162570 (ChIP-seq), and GSE190424 (single cell ATAC-seq).

Field-specific reporting

Please select the one below that is the best fit for your research. If you are not sure, read the appropriate sections before making your selection.

- [x] Life sciences
- [ ] Behavioural & social sciences
- [ ] Ecological, evolutionary & environmental sciences

For a reference copy of the document with all sections, see nature.com/documents/nr-reporting-summary-flat.pdf

Life sciences study design

All studies must disclose on these points even when the disclosure is negative.

Sample size	Sample size was determined based on previous similar studies (Sun et al., 2014, Wahlestedt et al., 2013)<br>In the ATAC analysis, we increased the number of HSC samples to detect DARs more precisely.
Data exclusions	All sequence data were included in this analysis.
Replication	large portion of HSC DEGs were shared with previous reports(Sun et al., 2014)(Wahlestedt et al., 2013) (Figure 1F, Table S1), and the results of ATAC-seq are integrated with RNA-seq and other epigenome (H3K27ac, H3K4me, DNA-methylation) to validate the result.<br>t-SNE was tried with perplexity 7,8,9,10 and iteration 3000,4000,5000,6000 respectively, and all results were consistent.
Randomization	No randomization was used because we compared young C57BL/6 and aged C57BL/6, which are isogenic.
Blinding	The researcher was not blinded.<br>Given that gating for cell sorting and data processing of NGS were performed automatically, the subjectivity of the author would not have affected the analysis.

Reporting for specific materials, systems and methods

We require information from authors about some types of materials, experimental systems and methods used in many studies. Here, indicate whether each material, system or method listed is relevant to your study. If you are not sure if a list item applies to your research, read the appropriate section before selecting a response.

Materials & experimental systems

n/a	Involved in the study	
[ ]	[x]	Antibodies
[x]	[ ]	Eukaryotic cell lines
[x]	[ ]	Palaeontology and archaeology
[ ]	[x]	Animals and other organisms
[x]	[ ]	Human research participants
[x]	[ ]	Clinical data
[x]	[ ]	Dual use research of concern

Methods

n/a	Involved in the study	
[x]	[ ]	ChIP-seq
[ ]	[x]	Flow cytometry
[x]	[ ]	MRI-based neuroimaging

Antibodies

Antibodies used	Rat anti-mouse c-Kit-APC (clone 2B8) BioLegend Cat.# 105812 (1:200 dilution)<br>Rat anti-mouse CD150-PE (clone TC15-12F12.2) BioLegend Cat.# 115904 (1:100 dilution)<br>Rat anti-mouse CD135-BV421(clone A2F10) BioLegend Cat.# 135314 (1:200 dilution)<br>Rat anti-mouse CD34-FITC (clone RAM34) eBioscience Cat.# 11034185 (1:50 dilution)<br>Rat anti-mouse Sca1-PE/Cy7 (clone D7) BioLegend Cat.# 108114 (1:200 dilution)<br>Rat anti-mouse CD48-APC/Cy7 (clone HM48-1) BioLegend Cat.# 103432 (1:200 dilution)<br>Streptavidin-PerCP/Cy5.5 BioLegend Cat.# 405214 (1:200 dilution)<br>Rat anti-mouse Gr-1-biotin (clone RB6-8C5) TONBO/biosciences Cat.# 30-5931-U500 (1:200 dilution)<br>Rat anti-mouse Mac-1-biotin(clone M1/70) TONBO/biosciences Cat.# 30-0112-U500 (1:200 dilution)<br>Rat anti-mouse Ter119-biotin(clone TER-119) BioLegend Cat.# 116204 (1:200 dilution)<br>Rat anti-mouse B220-biotin(clone RA3-6B2) TONBO/biosciences Cat.# 30-0452-U500 (1:200 dilution)<br>Rat anti-mouse IL-7Rα-biotin (clone SB/199) BioLegend Cat.# 121104 (1:200 dilution)<br>Rat anti-mouse CD4-biotin (clone GK1.5) BioLegend Cat.# 100404 (1:200 dilution)<br>Rat anti-mouse CD8a-biotin (clone 53–6.7) TONBO/biosciences Cat.# 30-0081-U500 (1:200 dilution)<br>Rat anti-mouse CD135-PE (clone A2F10) BioLegend Cat.# 135306 (1:200 dilution)<br>Rat anti-mouse FcγR-PE (clone 93) BioLegend Cat.# 101308 (1:100 dilution)<br>Rat anti-mouse IL-7Rα-PE(clone A7R34) BioLegend Cat.# 135010 (1:100 dilution)

Validation	Combination of these antibodies were already utilized by many published articles.<br>Nat Commun 12(1):3568, 2021.<br>J Exp Med 218(3):e20192283, 2021.<br>Leukemia 35:1156-1165, 2021.<br>Blood 128(5):638-649, 2016.<br>Exp Hematol 44:282-296, 2016.<br>Blood 126:1172-1183, 2015<br><br>All antibodies are validated by company. Validation are noted in technical data sheet of product pages, listed as follows<br>https://www.biolegend.com/ja-jp/products/apc-anti-mouse-cd117-c-kit-antibody-72<br>https://www.biolegend.com/ja-jp/products/pe-anti-mouse-cd150-slam-antibody-1369?GroupID=BLG10572<br>https://www.biolegend.com/ja-jp/products/brilliant-violet-421-anti-mouse-cd135-antibody-8728?GroupID=BLG7934<br>https://www.fishersci.com/shop/products/cd34-rat-anti-mouse-fitc-clone-ram34-ebioscience-1/509532<br>https://www.biolegend.com/ja-jp/products/pe-cyanine7-anti-mouse-ly-6a-e-sca-1-antibody-3137<br>https://www.biolegend.com/ja-jp/products/apc-cyanine7-anti-mouse-cd48-antibody-8054?GroupID=BLG6848<br>https://www.biolegend.com/ja-jp/products/percp-cyanine5-5-streptavidin-4212<br>https://tonbobio.com/products/biotin-anti-mouse-ly-6g-gr-1-rb6-8c5<br>https://tonbobio.com/products/biotin-anti-human-mouse-cd11b-m1-70<br>https://www.biolegend.com/ja-jp/products/biotin-anti-mouse-ter-119-erythroid-cells-antibody-1864<br>https://tonbobio.com/products/biotin-anti-human-mouse-cd45r-b220-ra3-6b2<br>https://www.biolegend.com/ja-jp/products/biotin-anti-mouse-cd127-il-7ralpha-antibody-3048<br>https://www.biolegend.com/ja-jp/search-results/biotin-anti-mouse-cd4-antibody-247<br>https://tonbobio.com/products/biotin-anti-mouse-cd8a-53-6-7<br>https://www.biolegend.com/ja-jp/products/pe-anti-mouse-cd135-antibody-6173<br>https://www.biolegend.com/ja-jp/products/apc-cyanine7-anti-mouse-cd117-c-kit-antibody-5905?GroupID=BLG4276<br>https://www.biolegend.com/ja-jp/products/pe-anti-mouse-cd16-32-antibody-189<br>https://www.biolegend.com/ja-jp/products/pe-anti-mouse-cd127-il-7ralpha-antibody-6190?GroupID=BLG7953

Animals and other organisms

Policy information about studies involving animals; ARRIVE guidelines recommended for reporting animal research

Laboratory animals	Ten-week-old female C57BL/6 mice (B6-CD45.2) were purchased from Japan SLC (Japan) and bred for 16–19 months in the animal experiment facilities of Chiba University and The Institute of Medical Science, The University of Tokyo (IMSUT).<br>Housing conditions: temperature 22 ± 2°C, humidity 55 ± 5%, light/dark cycle 12hour/12hour (8am-20pm light).
Wild animals	Not applicable. No wild animals were used in this study.
Field-collected samples	Not applicable. No Field-collected samples were used in this study.
Ethics oversight	All experiments using mice were performed in accordance with our institutional guidelines for the use of laboratory animals and approved by the Review Board for Animal Experiments of Chiba University (approval ID: 30–56) and IMSUT (approval ID: PS18–02)

Note that full information on the approval of the study protocol must also be provided in the manuscript.

Flow Cytometry

Plots

Confirm that:

- [x] The axis labels state the marker and fluorochrome used (e.g. CD4-FITC).
- [x] The axis scales are clearly visible. Include numbers along axes only for bottom left plot of group (a 'group' is an analysis of identical markers).
- [x] All plots are contour plots with outliers or pseudocolor plots.
- [x] A numerical value for number of cells or percentage (with statistics) is provided.

Methodology

Sample preparation	Bone Marrow cells were isolated by crashing bones from the back bone, pelvis, femurs, and tibiae. Cells were incubated with a mixture of biotin-conjugated monoclonal antibodies against lineage (Lin) markers s including Gr-1, Ter-119, B220, CD4, CD8, and IL-7Rα (SB/199). Cells were stained further with fluorochrome-conjugated streptavidin and a combination of antibodies
Instrument	FACSAria III,FACSCelesta (BD)
Software	FlowJo v10
Cell population abundance	Cell sorting efficiency was confirmed by flow cytometric analysis of post-sorted cells

Gating strategy

FSC-A/SSC-A were used for mononuclear cells gating. FSC-H/FSC-W were used for single cell gating.
Live cells were gated by PI-. Then Lin- cells were gated, and c-Kit+/Sca1+ population was defined as LSK
CD34-FITC negative cells were gated, and CD150+/CD48- gating population was defined as hematopoietic stem cells.

☒ Tick this box to confirm that a figure exemplifying the gating strategy is provided in the Supplementary Information.
